# Theoretically designed M@diaza[2.2.2]cryptand complexes: the role of non-covalent interactions in promoting NLO properties of organic electrides

**DOI:** 10.1080/14686996.2024.2357064

**Published:** 2024-05-20

**Authors:** Atazaz Ahsin, Aamna Qamar, Qing Lu, Wensheng Bian

**Affiliations:** a Beijing National Laboratory for Molecular Sciences, Institute of Chemistry, Chinese Academy of Sciences, Beijing, China; b School of Chemical Sciences, University of Chinese Academy of Sciences, Beijing, China; c Beijing National Laboratory for Molecular Sciences, State Key Laboratory of Polymer Physics and Chemistry, Chinese Academy of Sciences, Beijing, China

**Keywords:** Electrides, excess electrons, optoelectronic, molecular dynamics, DFT

## Abstract

Organic excess electron compounds with significant nonlinear optical (NLO) properties are widely employed in optoelectronic applications. Herein, single-alkali metals with diaza[2.2.2] cryptand (M@crypt,M=Li, Na, and K) are investigated for optoelectronic and NLO properties by using the density functional theory. Thermodynamic and kinetic stabilities of present complexes are computed through interaction energy (E_int_) and *ab-initio* molecular dynamic (AIMD) simulations. M@crypt complexes carry excess electrons and mimic molecular electrides. Quantum theory of atoms in molecules (QTAIM) analysis and reduced density gradient (RDG) spectra demonstrate the roles of the weak van der Waals (vdW) interactions between metal and complexant. The remarkable hyperpolarizability (β_o_) value up to 1.41 × 10^6^ au may be credited to the presence of loosely bound excess electrons. The hyper Rayleigh scattering hyperpolarizability (β_HRS_) is recorded up to 1.31 × 10^6^ au for the K@crypt. Furthermore, frequency-dependent first-order and second-order hyperpolarizability is more prominent at the applied frequency of ω = 0.042823 au. The electron localizing function (ELF) and localized orbital locator (LOL) analysis further disclose the nature of interaction between alkali metal and complexant. The TD-DFT method is adopted to get excited state parameters and absorbance properties. An electron density difference map (EDDM) is exploited to evaluate the orbital contributions in excited states. Hence, the studied electride may become a promising candidate for NLO materials. We anticipate that the present work will provide insight into further development of molecular electride for optoelectronic applications.

## IMPACT STATEMENT

Organic electrides M@cryptand [2.2.2] (where M=Li, Na, and K) are examined as excess electrons, where vdW forces play important roles in promoting the NLO properties.

## GRAPHICAL ABSTRACT

**Figure:**
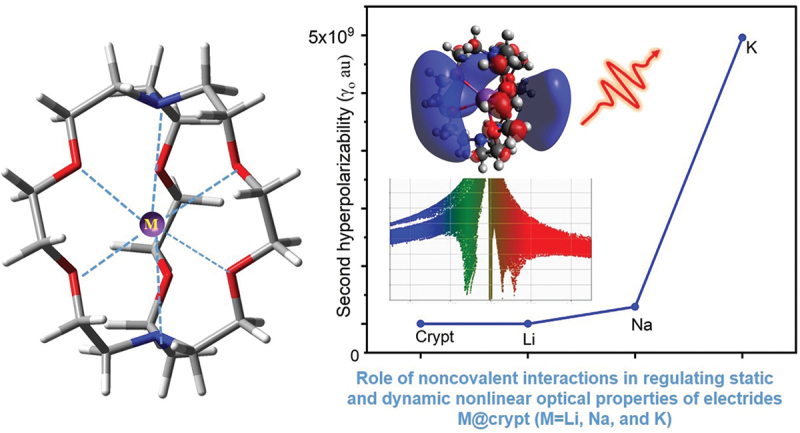

## Introduction

Nonlinear optical (NLO) materials have been extensively used in the fields of fibre optics, data transformation, photonic lasers, and data storage in wireless communication due to the recent advancement in optics [1–3]. After the discovery of the first working ruby laser by Maiman in 1960, optics became a more intense subject in interdisciplinary science [4]. Several materials have demonstrated excellent optical nonlinearity in recent decades, including organic materials [5], perovskite quantum dots [6], nanoclusters [7], and organic-inorganic hybrid materials [8,9]. Therefore, scientists are focusing on the development of novel organic NLO materials to establish future society. Numerous organic crystals, such as 4-dimethylamino-N-methyl-4-stilbazolium-tosylate (DAST) [9], N-benzyl-2-methyl-4-nitroaniline (BNA) [10], and 2,7-di(pyridine-3-yl)-9 H-flupren-9-one (3-DPyFO) and 2,7-di(pyridine-4-yl)-9 H-flupren-9-one (4-DPyFO), have been found to exhibit the NLO effect [10].

Asymmetric charge distribution [11], significant dipole moment (µ_o_), and low excitation (ΔE) energy, are considered crucial factors in enhancing the NLO response of molecular materials [12,13]. Up till now, various kinds of measures have been considered to improve the molecular structure for promoting the optoelectronic at the molecular level. Among the adopted strategies, constructing donor-conjugated-bridge-acceptor (D-A) models [14], introducing push-pull effects [15], making sp^2^-hybridized carbon-based materials [16], designing multidecker sandwiches complexes [17], creating octupolar molecules [18], bond length alternation (BLA) theory [19], and introduction of loosely bound electrons (excess electrons) are very renowned for constructing NLO materials [20,21]. Among the above-stated techniques, investigating excess electron molecules is perhaps the most fascinating way to enhance the NLO properties. The doping of transition and main group metals [on host molecules] is an emerging approach for introducing excess electrons into materials [22,23]. Research has shown that the existence of loosely bound electrons greatly reduces the excitation energy (ΔE), facilitates electron promotion to unoccupied orbitals, and is required for regulating the optical and NLO characteristics [24–26].

Alkalides (alkali anion) [27], alkaline earth metals earthides [28], and electrides [23] are the main classes of excess electron compounds and were studied for various applications. In alkalides, the alkali metals act as anions (Li^−^, Na^−^ and K^−^) for charge transfer, while the 2nd group (earth metals) contains anionic nature in earthides. Theoretically designed alkalides were reported using the alkali metals and superalkali clusters with various complexant [27,29]. In such complexes, the role of alkali metal is to transfer charge, and strong polarization was seen. For instance, Ayub *et al.* [29] have designed an alkalides Li_3_O@[12-crown-4]M (where M=Li, Na, and K) as excess electrons complexes by simultaneous doping of Li_3_O and alkali metals on the crown ether. Similarly, a variety of diffuse excess electron compounds were designed for NLO applications based on cages and crown ether complexes [30,31]. Electrides containing excess electrons (diffuse excess) in lattice voids as anions have grabbed considerable attention in both fundamental research and application development [32]. The very first evidence about the synthesis of electride was received in 1983 by Dye and co-workers. Since then, numerous organic and inorganic electrides have been reported [33,34]. Johnson and colleagues investigated eight organic electrides through a DFT study and the presence of localized interstitial electrons justifies their electrides properties [35]. They also claimed the strong adsorption affinity Na^+^ through complexation with cryptand (crypt), containing eight tertiary amine nitrogen [36]. Owing to the excess electron nature of electride, they are continuously considered to construct remarkable NLO materials. Very recently, Wajid *et al.* theoretically designed alkali metals-doped C_6_O_6_Li_6_ organometallic electrides for optical and NLO applications [37]. For the K@C_6_O_6_Li_6_ complex, the hyperpolarizability (β_o_) value was recorded up to 2.9 × 10^5^ au. Likewise, M_3_O@C_6_S_6_Li_6_ (*M* = K, Na, Li) electrides were also reported for geometric, electronic, and optical properties [38].

Cryptands belong to the family of synthetic bi- and polycyclic organic compounds and have received great attention due to their excellent structural feature. Cryptand-based hosts are very famous for unique three-dimensional structures, which can be exploited in constructing novel complexes [39,40]. In 1987, Lehn and their co-workers [41] successfully synthesized cryptand for the first time. The interior cavity can be operational for the sitting of guest molecules and atoms in modifying its properties. Using the cavity of benzocryptand, Nimra *et al.* have reported alkali metals doped benzocryptand for NLO application, where hyperpolarizability response was recorded up to 9.1 × 10^5^ au [42]. The evidence of the strong interaction of alkali metal cations with cryptand and their trapped electrons (electride) has already been emphasized [43]. The dye group has experimentally reported thermally stable K^+^(cryptand 2.2.2)e^−^ electride, where ionic solids with cavity-trapped electrons serve as the anions [44,45].

**Figure 1.**
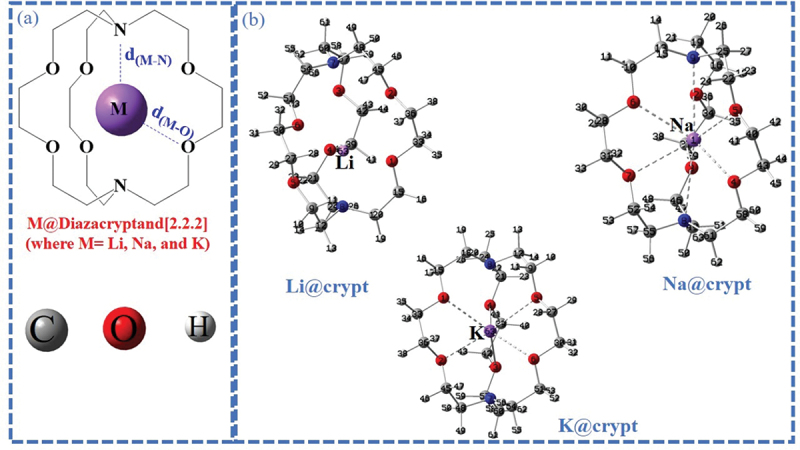
Representation of optimized labeled geometries (a) pure diazacryptand [2.2.2] and (b) M@crypt complexes using the ωB97×d/def2-tzvp level of theory.

Inspired by earlier developments in molecular electrides and their NLO properties, we theoretically designed single alkali metal-based electrides based on diazacryptand [2.2.2]. We aim to uncover the electride nature, and role of noncovalent interactions in promoting optoelectronic properties of M@crypt (where M=Li, Na, and K) complexes (Figure 1). It should be noted that the van der Waals (vdW) interaction is a kind of important noncovalent interactions, which plays very important roles in many chemical processes [46,47], and includes dispersion interaction as well as others. Its role in the formation and properties of molecular complexes is of great interest.

## Computational methodology

The ground state structures of the designed M@crypt were fully optimized with the ωB97×d/def2-tzvp level of theory. The entire simulations were performed using the Gaussian 16 and ORCA 5.0 package [48,49]. The long-range corrected method (ωB97×d) yields satisfactory results for thermochemistry, adsorption study, and non-covalent interactions [50]. Interaction energies are calculated at the same method to justify the thermodynamic stability of present complexes. Additionally, we have performed *ab-initio* molecular simulation (AIMD) at 300 K to assess the kinetic and thermodynamic stability using the ORCA 5.0 program. Results of AIMD comprise 1500 distinct geometries in each trajectory of the complex, which are adopted for further analysis. Furthermore, AIMD is executed for 750 femtoseconds (fs) with a step size of 0.5 using the B3LYP-D3/def2-SVP method [44]. The adsorption nature of complexes is determined through calculated interaction energy (E_int_), which is given by the following relation;

(1) $$E_{\mathrm{int}}=E\left(M@\mathrm{crypt}\left[2.2.2\right]\right)-\left\{E\left(M\right)+E\left(\mathrm{crypt}\left[2.2.2\right]\right)\right\}\left(\mathrm{where}\;M=\mathrm{Li},\mathrm{Na},\mathrm{and}\;K\right)$$

The charge transfer study, electride character, and orbital energies are also calculated through NBO and frontier molecular orbital (FMO) theory. Vibrational frequencies are computed using the same method, where the mode of vibrations is compared with the experimental study. Vertical ionization potential (IP), electron affinity (EA), and hardness (*η*) were computed for the present complexes. These parameters are given by the following equations [51,52];

(2) $$\mathrm{Vertical}\;\mathrm{Ionization}\;\mathrm{potential}\;\left(\mathrm{VIP}\right)\;=\;E_{{\left(\mathrm{COMPLEX}\right)}^{+}}-E_{{\left(\mathrm{COMPLEX}\right)}^{0}}$$

(3) $$\mathrm{Vertical}\;\mathrm{electron}\;\mathrm{affinity}\;\left(\mathrm{EA}\right)\;=\;E_{{\left(\mathrm{COMPLEX}\right)}^{-}}-E_{{\left(\mathrm{COMPLEX}\right)}^{0}}$$

(4) $$\eta ={\left(\frac{\partial^{2}E}{\partial N^{2}}\right)}_{V\left(r\right)}$$

The optical and nonlinear optical (NLO) properties were characterized through calculated values of polarizability (α_o_), hyperpolarizability (β_o_), and projection of hyperpolarizability on dipole moment (β_vec_) using the ωB97×d/def2-tzvp level of theory. A comparison of static NLO properties is made using the CAM-B3LYP and M062X, and ωB97×d method with def2-tzvp basis set. Scattering hyperpolarizability values are calculated for hyper Rayleigh scattering measurement at the ωB97×d/def2-tzvp level of theory. The effect of solvent on NLO response is also considered using the same method. Frequency-dependent (dynamic) first-order hyperpolarizability β(ω) and second-order hyperpolarizability γ(ω) were calculated at the applied frequencies of 532 and 1064 nm using ωB97×d/def2-tzvp at which frequencies experimental studies were carried out.

Perturbation theory may be used to calculate the energy and nonlinear optical parameters. The Taylor expansion describes the energy of the perturbed system.

(5) $$E=E^{O}-\mu_{I}F_{i}-\left[\frac{1}{2!}\right]\alpha_{\mathrm{ij}}F_{i}F_{j}-\left[\frac{1}{3!}\right]\beta_{\mathrm{ijk}}F_{i}F_{j}F_{k}-\left[\frac{1}{4!}\right]\gamma_{\mathrm{ijkl}}F_{i}F_{j}F_{k}F_{l}$$

(6) $$\mu =\sqrt{{\mu^{2}}_{X}+{\mu^{2}}_{Y}+{\mu^{2}}_{Z}}$$

$$\alpha_{\mathrm{ij}}={\left(\frac{\delta \mu_{i}}{\delta F_{j}}\right)}_{E_{\to 0}}$$

(7) $$\alpha_{\mathrm{tot}}=\frac{1}{3}\left(\alpha_{\mathrm{xx}}+\alpha_{\mathrm{yy}}+\alpha_{\mathrm{zz}}\right)$$

The magnitude of hyperpolarizability can be defined as;

$$\beta_{x}=\beta_{\mathrm{xxx}}+\beta_{\mathrm{xyy}}+\beta_{\mathrm{xzz}}$$

$$\beta_{y}=\beta_{\mathrm{yyy}}+\beta_{\mathrm{yxx}}+\beta_{\mathrm{yzz}}$$

$$\beta_{x}=\beta_{\mathrm{zzz}}+\beta_{\mathrm{zxx}}+\beta_{\mathrm{zyy}}$$

(8) $$\beta_{\mathrm{tot}}=\sqrt{{\beta^{2}}_{x}+{\beta^{2}}_{y}+{\beta^{2}}_{z}}$$

(9) $$\gamma =\gamma_{\mathrm{tot}}=\frac{1}{5}\left(\gamma_{\mathrm{xxxx}}+\gamma_{\mathrm{yyyy}}+\gamma_{\mathrm{zzzz}}+2\gamma_{\mathrm{xxyy}}+2\gamma_{\mathrm{xxzz}}+2\gamma_{\mathrm{yyzz}}\right)$$

Hyper-Rayleigh scattering (β_HRS_) response is described as [53];

(10) $$\beta_{\mathrm{HRS}}\left(0;0,0\right)=\sqrt{\left\{\left({\beta_{\mathrm{ZZZ}}}^{2}\right)\right\}+\left\{\left({\beta_{\mathrm{ZXX}}}^{2}\right)\right\}\;}$$

The β_HRS_ is composed of dipolar φ(β_J *=* 1_) and octupolar φ(β_J *=* 3_) contribution to total hyperpolarizability and given by [54];

$${\beta_{\mathrm{ZZZ}}}^{2}=\frac{9}{5}{\left|\beta_{J=1}\right|}^{2}+\frac{6}{105}{\left|\beta_{J=3}\right|}^{2}$$

(11) $${\beta_{\mathrm{ZXX}}}^{2}=\frac{1}{45}{\left|\beta_{J=1}\right|}^{2}+\frac{4}{105}{\left|\beta_{J=3}\right|}^{2}$$

To unfold the nature of the interaction between metal and crypt, topology analysis was exploited using Bader’s quantum theory of atoms in molecules (QTAIM) and reduced density gradient (RDG) spectra [55]. In this analysis, we considered bond critical points (BCP) at (3,-1) electron density. Electron density difference maps are also obtained with the same method. An excited state analysis was carried out using TD-ωB97×d/def2-tzvp to get the absorbance pattern, excitation energy, and orbital contributions during the crucial transition. The projected density of states (PDOS) spectra were plotted using GaussSum and multiwfn software [56]. In addition, the electron localizing function (ELF) and localized orbital locator (LOL) are plotted to understand the bonding properties in present complexes. The kinetic energy density plays a vital role in the description of a chemical bond, since the driving force in forming a covalent bond. The ELF function can nicely unveil the location of atomic shells, core binding electrons, and lone pairs electrons in atomic and molecular orbitals. Mathematically, the ELF can be expressed based on kinetic-energy density [57];

(12) $$\mathrm{ELF}={\left(1+{x^{2}}_{\sigma }\right)}^{-1}$$

(13) $$\mathrm{Where}\;\chi_{\sigma }=\frac{D_{\sigma }}{{D_{\sigma }}^{0}}\;\mathrm{and}\;D_{\sigma^{0}}=\frac{5}{3}{\left(6\pi^{2}\right)}^{2/3}{\rho_{\sigma }}^{5/3}$$

Where $D_{\sigma^{0}}$ is uniform electron gas with a spin density equal to the local value of *ρ*_*σ*_ (r) χ_σ_ ratio is a dimensionless quantity, indicating localization index and calibrated by considering uniform-density electron gas as reference.

(14) 0≤ELF≤1

The possible ELF values range with the upper limit ELF = 1, indicating perfect localization and the value of ELF = 0.5 represents the electron gas-like pair probability.

On the other hand, the LOL is similar to the ELF, where just the orbital kinetic energy density and the local spin density approximation (LSDA) kinetic energy density are compared, without considering the Pauli repulsion [58]:

(15) $$\mathrm{LO}L_{\sigma }=\frac{1}{1+\frac{2t_{\sigma }}{{t_{\sigma }}^{\mathrm{LSDA}}}}$$

## Results and discussion

### Electronic structure and energetic analysis of complexes

The optimized geometries of M@crypt (M=Li, Na, K, and crypt = diazacryptand[2.2.2]) complexes using ωB97×d/def2tzvp are shown in Figure 1. The adsorbed alkali metals have unequal interaction distances from bridgehead nitrogen atoms (d_M-N_) and with the oxygen of the cyclic ring (d_M-O_). The interaction of alkali metals with nitrogen and oxygen is crucial to characterize the physico-chemical properties of complexes. Only influential bond distances were computed and values are given in supplementary information (Table S1). In a reported study on the same system, the geometries of K@crypt and Na@crypt complexes were slightly distorted from the ideal D_3_-group [59]. In the present results, a similar scenario is seen except for a slight distortion in Li@crypt, where Li is asymmetrically adsorbed in the cavity. In Li@crypt, the calculated bond distances between Li63-N7 and Li63-N8 are 3.95 and 2.46 Å. The Li-atom has a higher distance with N7 while strongly interacting with lower bridgehead nitrogen (N8). Similarly, average optimized distance of Li-atom with oxygen (d_M-O_) is 2.34 to 2.70 Å. For Na@crypt, the interaction distance between the Na1-N8/N9 is 3.19 Å (Table S1). Similarly, Na1 is equidistant from O4/O5 calculated up to 2.48 Å. The interaction distance of K63- N7 and K63-N8 is 3.07 Å (see Table S1). K-metal is adsorbed exactly in the middle of the cavity and is equidistant from nitrogen (N7, and N8) and oxygen of the ring. The obtained structural results were also compared with the reported experimental synthesis of (2.2.2-cryptand)potassium nitrate hydrate complex [60], and values are given in supplementary information (Table S1). The K-O interaction distance reported by Boldyrev et al. [59] has a comparable value to the present results (see Table S1). There is no imaginary frequency associated with complexes, denoting their stability on potential energy surfaces.

The obtained significant interaction energies (E_int_) quantify their thermodynamic stability (see Table 1). The interaction energies range from -11.10 to -21.91 kcal/mol, where the highest value is obtained for Li@crypt, while the lowest is calculated for Na@crypt. Overall, the E_int_ of present electrides are higher in comparison with the reported electride, evidencing the strong interaction and formation of stable complexes. The significant E_int_ of Li@crypt is in accordance with the least interaction distances of Li with N7 (2.46 Å) and oxygen atoms (2.70 Å). The E_int_ (-21.91 kcal/mol) of present Na@crypt is higher than alkali-doped benzocryptand [42], superalkalis doped all-cis-1,2,3,4,5,6-hexachlorocyclohexane [61], and M_3_O@C_6_S_6_Li_6_ (*M* = K, Na, Li) electrides.

**Table 1.** Interaction energies (E_int_ kcal/mol), vertical ionization potential (VIP eV), vertical electron affinity (VEA eV), chemical hardness (Ƞ eV), HOMO and LUMO energies (in eV), energy gap (E_H-L_ eV), NBO charge on alkali metals (QM |e|), NBO charge in nitrogen (QN |e|), and highest NBO charge on oxygen (QO |e|) of complexes.

	E_int_	VIP	VEA	Ƞ	E_HOMO_	E_LUMO_	E_H-L_	Q(M)	Q(N)	Q(O)
Crypt	N/A	7.56	3.14	4.42	-7.60	-3.18	4.46	N/A	-0.42	-0.49
Li@Crypt	-21.91	1.90 2.05^a^	2.01	0.47	-2.12	2.59	0.47	0.04	-0.65	-0.63
Na@Crypt	-11.10	1.01 1.96^a^	1.43	0.38	-1.17	1.55	0.38	0.01	-0.60	-0.64
K@Crypt	-20.56	1.08 1.95^a^	0.80	1.07	-1.14	2.21	1.06	-0.0001	-0.58	-0.65

[a] Values were reported using the PBE0/6–311++G** method [61].

A comparison of the vibrational study of the pure complexant (diazacryptand) and alkali metal-encapsulated complexes is made with reported FT-IR analysis to monitor changes in vibrational modes. The vibrational frequency and their comparison with the experimental values are summarized in supplementary information (Table S2). For the crypt, the intense peaks of C-H stretching vibrations were observed in the range of 2812–2981 cm^−1^. C-H bending peaks range from 1280 to 1523 cm^−1^ and are second-strong peaks. The experimental value of C-H stretching ranges from 2790 cm^−1^ to 2877 cm^−1^ (see Table S2) [62]. Further details of characteristics IR peaks is given in supplementary information (see section S2).

*Ab-initio* molecular dynamics (AIMD) calculations of designed complexes were performed using ORCA 5.0.2, time step 0.5 (fs), and at room temperature (300 K). AIMD results show that M@crypt remained stable during simulation, with rigid structures against isomerization and decomposition. The root-mean-square deviation (RMSD) for complexes and pure crypt is plotted and given in supplementary information (see Figure S1). RMSD is a standard measure of structural distance between coordinates. The variation in kinetic energy versus time is also plotted in Figure S1b. In the beginning, kinetic energy increases for complexes up to 200 fs while equilibrium is attained after 300 fs. Additionally, the drift energy (K) versus time (fs) spectra were also given in supplementary information (Figure S2). The drift energy (K) of Li@crypt is higher in the beginning and near 800 fs. Likewise, the drift energy for Na@crypt is maximum near 200 fs and noticeably reduced at 700 fs. The snapshots of geometries after 100 fs are captured to ensure the rigidity of complexes against dissociation and are shown in the supplementary information (Figure S3). The curves of complexes have an uptrend with the increased size of metals (Li to K). Hence, the AIMD study reveals their thermodynamic and kinetic stability at room temperature.

**Figure 2.**
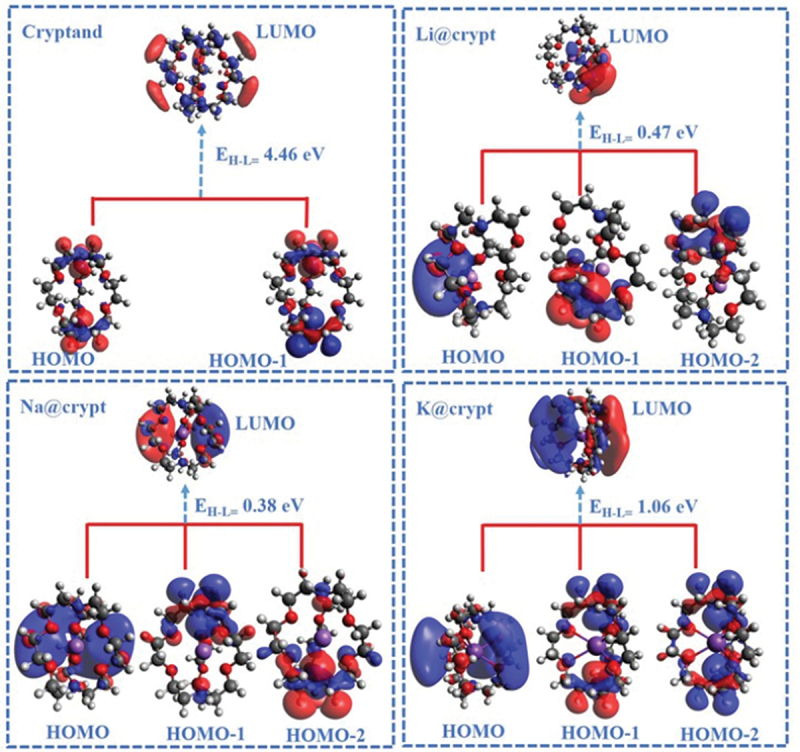
3D representation of frontier molecular orbitals (FMOs) involved in electronic excitations of designed complexes using the ωB97×d/def2-tzvp method. (isovalue = 0.015).

The computed partial NBO charges are given in Table 1. The calculated charges on alkali metals Q(M) are slightly positive and range from 0.40 to -0.0001 |e|. In Li@crypt, the small Li to nitrogen distance (d_M-N_) causes significant charge transfer (due to strong interaction) to nitrogen (N7), which ranges from -0.65 to -0.57 |e|. Similarly, for the Na@crypt complex, slightly reduced NBO charge (positive) on Na-atom up to + 0.013 |e| while, the K-atom shows negligible positive NBO charge. Due to the interaction of metals with cryptand, ns^1^ electrons are polarized by neighbour atoms (N and O) of complexant. The lowest polarization of partial charge is observed on the K-atom, attributing to symmetric adsorption of K-metal inside cavity. One can conclude that loosely bound electrons are trapped inside the complexant and induce the electride nature to complexes. The electride nature and charge transfer predominately enhance the optical properties, which will be shown later. Further justification of electride nature and orbital energies can be established through frontier molecular orbital (FMO) analysis (see section S3). The orbital shapes of present complexes are given in Figure 2. The HOMO is not warping a particular atom distributed in space in the complexant. After the alkali-metals encapsulation, these complexes have HOMO and LUMO in space distributed within the entire complexant, which is reminiscent of the molecular electride. The HOMO has diffused shape and indicates excess electrons are trapped inside the complex. Vertical ionization potential (VIP), electron affinity (VEA), and chemical hardness (η) are also calculated and values are given in Table 1. The VIP of the pristine crypt is 7.56 eV, while its VEA is 3.14 eV. After the interaction of alkali metals (Li, Na, and K), a significant reduction in VIP and VEA values is observed. The calculated VIP values of complexes are 1.90, 1.01, and 1.08 eV for Li@crypt, Na@crypt, and K@crypt, respectively. VIP values are endowed to be useful to manifest their capability to donate and accept electrons. The reduced VIP values with increased alkali metals size (Li to K) suggest the electropositivity and excellent reducing ability of present complexes. Smaller ionization potential than Cs-atom (3.87 eV) also suggests their superalkali-like nature [63]. The VIP, VEA, HOMO-LUMO gaps, and interaction energies are compared with similar reported complexes and values are given supplementary information (Table S3).

### Static nonlinear optical (NLO) properties of complexes

Owing to the electride nature of present complexes, one may envisage interesting NLO features. Various excess electron compounds were investigated as efficient NLO candidates, in which electrides are the most promising candidates [22,26,64–67]. NLO parameters of M@crypt (electrides) are given in Table 2. The dipole moment (µ_o_) plays a vital role in studying the polarization, packing, and asymmetric charge distribution. A notable value of µ_o_ can be correlated to higher NBO charges on and asymmetric interaction between metal (M) and cryptand. A remarkable µ_o_ of Na@crypt complex may be credited to the strong asymmetric interaction of metal Na1 with N7, N8, and oxygen atoms of the cryptand ring. The lowest value of µ_o_ can be seen in K@crypt, where K63 is exactly in the middle of the cryptand cavity. Static polarizability (α_o_) provides information on the distribution of electrons in a molecule and is critical in deciding the polar nature and reactivity of present complexes. The obtained (α_o_) value of pristine complexant is 2.54 × 10^2^ au, while after the interaction of alkali metals M@crypt, the *α*_*o*_ increased up to 9.30 × 10^2^ au (Table 2). Also, the z-component shows a significant contribution to the total polarizability response. The gradual increase in α_o_ is observed as follows: K@crypt >Li@crypt > Na@crypt. The values of α_o_ at M06-2X/def2qzvp and CAM-B3LYP/def2tzvp method are close to ωB97×d with same basis set and are provided in supplementary information (Table S4).

**Table 2.** Mean dipole moment (µ_o_ debye), polarizability and its components (α_o_ au), hyperpolarizability and its components (β_o_ au), and projection of hyperpolarizability on dipole moment vector (β_vec_ au).

	µ_o_	α_o_	α_x_	α_y_	α_z_	β_o_	β_x_	β_y_	β_z_	β_vec_
Crypt	0	2.54 × 10^2^	2.76 × 10^2^	2.42 × 10^2^	2.42 × 10^2^	1.02 × 10^2^	25.00	96.16	24.67	6.489
Li@crypt	4.69	3.47 × 10^2^	4.14 × 10^2^	3.38 × 10^2^	2.89 × 10^2^	5.32 × 10^3^	3.48 × 10^3^	6.45 × 10^2^	3.97 × 10^3^	3.59 × 10^3^
Na@crypt	6.04	8.66 × 10^2^	6.56 × 10^2^	5.01 × 10^2^	3.75 × 10^3^	1.41 × 10^6^	2.57 × 10^4^	1.19 × 10^6^	7.64 × 10^5^	1.19 × 10^6^
K@crypt	0.10	9.30 × 10^2^	6.12 × 10^2^	1.10 × 10^3^	1.07 × 10^2^	9.83 × 10^5^	2.41 × 10^5^	9.50 × 10^5^	5.56 × 10^4^	2.96 × 10^5^

On the other hand, the hyperpolarizability (β_o_) values of complexes are 5.32 × 10^3^, 1.41 × 10^6^, and 9.30 × 10^5^ au for Li@crypt, Na@crypt, and K@crypt, respectively, where the highest value is observed for the Na@crypt complex (Table 2). The calculated significant value of 1.41 × 10^6^ is higher than that of the organic reference molecule (P-nitroaniline). Likewise, the computed β_o_ value of 1.41 × 10^6^ au for Na@crypt is quite higher than those of previously reported M@C_6_O_6_Li_6_ electrides, superalkali@Al_12_P_12_ electrides [37,68]. The non-monotonic trend of β_o_ for the Na@crypt complex using ωB97×d/def2tzvp might be attributed to its high dipole moment and low excitation energy (1.22 eV). Also, the asymmetric interaction has significantly altered the β_o_ response in Na@crypt. The β_o_ response of designed electrides can be correlated to electronic properties like VIP, EA, and chemical hardness. In the current study, lower VIP values indicate an increased β_o_ response. For instance, the highest β_o_ value of Na@crypt has the lowest VIP value (1.0), followed by K@crypt, where the VIP value (1.08 eV) is slightly higher using ωB97×d/def2-tzvp. A similar trend in values of projection of hyperpolarizability on dipole moment vector (β_vec_) and total hyperpolarizability (β_o_) can be seen which indicates excellent nonlinear optical properties of present complexes. The static second-order hyperpolarizability (γ_o_) is considered to be an important index to measure nonlinear optical coefficients of designed electrides. These values are calculated using ωB7XD/def2-tzvp and given in Table 3. The γ_o_ values are 1.05 × 10^4^, 4.30 × 10^5^, 2.96 × 10^8^, and 4.96 × 10^9^ au, respectively for crypt, Li@crypt, Na@crypt, and K@crypt. A gradual increase in γ_o_ can be seen with increased size of alkali metals (see Table 3). In contrary to hyperpolarizability, γ_o_ responses exhibit a dependence on the size of alkali metals. The obtained significant *γ*_*o*_ value of 4.96 × 10^9^ au for K@crypt is quite higher than previously reported alkali metal doped C_6_O_6_Li_6_ organometallics electride, superalkalis doped C_6_O_6_Li_6_ electrides [69], superalkalis@Al_12_P_12_ inorganic electrides [68], and alkaline-earth-based alkaline salt electrides M-H_3_C_4_N_2_⋯Ca (M=H, Li, and K) [70]. Observed β_o_ and γ_o_ values of M@crypt were significantly higher as compared to alkali and alkaline earth metal-based benzocryptand, superalkali@C_6_F_6_H_6_ electride, and Li_3_@C_60_ electride. Table S4 reveal the comparable β_o_ values at M062X and ωB97×d method, while values at CAM-B3LYP are slightly declined. A comparison of NLO parameters of present complexes is also made with reported similar molecular electrides and excess electron compounds using the ωB97×d method (Table S5).

**Table 3.** Static second hyperpolarizability (β_o_ au) scattering hyperpolarizability (β_HRS_ au), depolarization ratio (DR), percentage dipolar contribution to hyperpolarizability Φβ(j = 1), and octupolar contribution to hyperpolarizability Φβ(j = 3) of crypt and M@crypt complexes.

Complexes	γ_o_	γ_x_	γ_y_	γ_z_	β_HRS_	DR	Φβ(j = 1)	Φβ(j = 3)
Crypt	1.05 × 10^4^	6.9810^3^	*5.56*10^3^	*5.57*10^30^	170.21	1.500	0.1	99.9%
Li@crypt	4.30 × 10^5^	4.03 × 10^5^	1.45 × 10^5^	3.97 × 10^4^	1.33 × 10^4^	4.60	52%	48%
Na@crypt	2.96 × 10^8^	3.88 × 10^7^	2.92 × 10^8^	3.53 × 10^7^	9.01 × 10^5^	2.51	34%	66%
K@crypt	4.96 × 10^9^	1.04 × 10^8^	2.23 × 10^7^	4.94 × 10^9^	1.31 × 10^6^	3.53	44%	56%

The hyper-Rayleigh scattering (HRS) first-order hyperpolarizability (β_HRS_) of pure cryptand and electride complexes is also calculated using the same method. β_HRS_ is the most fundamental nonlinear chiral optical (chiroptical) effect to characterize the nonlinearity of molecular materials even with zero dipole moment. β_HRS_ is an important theoretical tool to measure the hyperpolarizability of centrosymmetric molecules [71]. The calculated values of β_HRS_, depolarization ratio (DR), dipolar contribution to hyperpolarizability (DR), percentage of dipolar contribution Φβ(j = 1) and octupolar contribution to total hyperpolarizability Φβ(j = 3) are given in Table 3. The undoped crypt holds a very low HRS (170.21) response as compared to M@crypt. For M@crypt, obtained β_HRS_ values are 1.33 × 10^4^, 9.0 × 10^5^, and 1.31 × 10^6^ au for Li@crypt, Na@crypt, and K@crypt, respectively. K@crypt is more responsive to HRS measurement, which can be correlated to the size of metal. The higher depolarization ratio (DR) indicates the dipolar nature of complexes. Pristine (crypt) shows the lowest DR ratio justifying its octupolar nature. The octupolar nature of the crypt is seen in its 99% contribution to hyperpolarizability at Φβ(j = 3). Complexes with a high percentage contribution from dipolar factor are characterized as dipolar (*vide supra*). The dipolar contribution to hyperpolarizability Φβ(j = 1) is almost 52% for Li@crypt, while Na@crypt and K@crypt complexes are octupolar due to significant contribution from Φβ(j = 3).

### Frequency-dependent (dynamic) NLO properties

The frequency-dependent hyperpolarizability β(ω) is calculated as β(-ω,ω,0) for electro-optical Pockel’s effect (EOPE) and β(-2ω, ω, ω) for second harmonic generation phenomena (SHG) parameter. Entire frequency-dependent NLO parameters are given in Table 4. To investigate the frequency-dependent NLO responses of the current complexes, we chose 532 and 1064 nm as transparent regions since the complexes display significant absorbance in between 700 nm and 1016 nm. The choice of wavelength (1064 nm) is also relevant to Nd:YAG laser functioning, which frequently emits invisible light in 1064 nm and serves in laser-based devices [72]. The electro-optic effect is the modification of a medium’s refractive index produced by an electric field. The electro-optical effect (Pockels effect) is an essential nonlinear effect used in many applications [73]. Only non-centrosymmetric materials (mostly nonlinear crystal materials) show the linear electro-optic effect, also known as the Pockels effect, in which the change in refractive index is proportional to the strength of the electric field. Overall, EOPE values are higher than those of electric field-induced second harmonic generation (ESHG) values at both frequencies. The EOPE values are 7.99 × 10^3^, 1.34 × 10^4^, 1.11 × 10^5^ au, respectively, for Li@crypt, Na@crypt, and K@crypt at larger frequency (ω = 0.085645). EOPE effect is most prominent at both frequencies. The highest value (9.55 × 10^7^ au) of EOPE is calculated for Na@crypt complex. The frequency-doubling nonlinear optical process is determined through ESHG. The ESHG values are almost comparable at both frequencies, while significantly lowered than those of EOPE. Frequency-dependent second-order hyperpolarizability γ(ω) is estimated using γ(-ω, ω,0,0) for dc-Kerr effect and γ(-2ω,ω,ω,0) as second harmonic generation phenomena (SHG). The computed parameters are given in Table 4. The Kerr effect, also known as the quadratic electro-optic (QEO) effect, is a change in a material’s refractive index in response to an applied electric field. The Kerr effect (electro-optic effect) reveals the nonlinear change in the refractive index of materials after externally applied fields. The nonlinear refractive index is given as [74];

**Table 4.** Frequency-dependent NLO properties; EOPE β(-ω, ω,0), ESHG β(-2ω,ω,ω), dc-Kerr effect γ(-ω, ω,0,0), and SHG γ(-2ω,ω,ω,ω) at 532 and 1064 nm.

	Frequency-dependent hyperpolarizability β(ω) using ωB97×d/def2-tzvp			
	532.37 nm (ω = 0.085645 au)		1064.73 nm (ω = 0.042823 au)	
	β(-ω, ω,0)	β(-2ω, ω, ω)	β(-ω,ω,0)	β(-2ω,ω,ω)
Crypt	0.60	0.64	0.590	0.60
Li@crypt	7.99 × 10^3^	4.99 × 10^4^	7.99 × 10^4^	2.98 × 10^4^
Na@crypt	1.34 × 10^4^	5.80 × 10^4^	9.55 × 10^7^	6.62 × 10^4^
K@crypt	1.11 × 10^5^	7.90 × 10^4^	7.50 × 10^3^	7.90 × 10^4^
	Frequency-dependent second hyperpolarizability γ(ω) using ωB97×d/def2-tzvp			
	γ(-ω, ω,0,0)	γ (-2ω,ω,ω,ω)	γ(-ω,ω,0,0)	γ (-2ω,ω,ω,0)
Crypt	1.20 × 10^4^	1.6 × 10^4^	1.0 × 10^4^	1.16 × 10^4^
Li@crypt	1.53 × 10^8^	1.85 × 10^7^	1.18 × 10^6^	6.26 × 10^6^
Na@crypt	2.48 × 10^8^	1.47 × 10^7^	7.36 × 10^12^	2.59 × 10^12^
K@crypt	2.83 × 10^7^	3.09 × 10^7^	2.70 × 10^7^	6.38 × 10

(16) $$\Delta n=\lambda {\mathrm{KE}}^{2}$$

where λ, K, and E are the wavelength of light, Kerr constant, and electric field strength, respectively. One can observe the higher Kerr effect takes place at a smaller frequency dispersion (ω = 0.042823 au). At 1064 nm, the highest dc-Kerr value of 7.36 × 10^12^ au is recorded for Na@crypt, while lowest is while lowest is Li@crypt (Table 4). The higher value of the Kerr effect also demonstrates a larger change in the refractive index of studied complexes. Overall, at both frequencies, the dc-Kerr γ(-ω, ω,0,0) beats SHG response.

### Role of non-covalent interactions in promoting NLO responses

**Figure 3.**
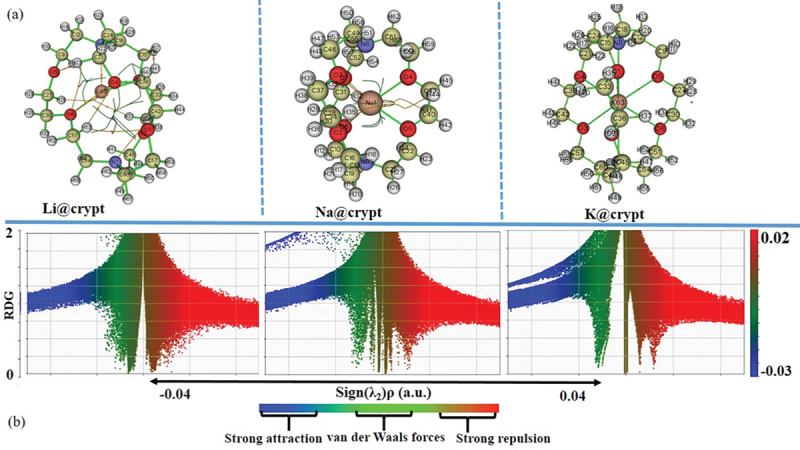
(a) Representation of labeled geometries for generated bond critical points (BCP) by considering the (3, -1) electron density, (b) (RDG) map of complexes using the ωB97×d/def2-tzvp level of theory.

Quantum theory of atoms in molecules (QTAIM) has been exploited to identify the bonding nature in M@crypt complexes. The presence of van der Waals (vdW) forces and other interactions are recognized as additional influencing factors in describing NLO properties of present complexes. Additionally, noncovalent interactions have crucial roles in describing chemical events and the stability of complexes in various organometallic and biological processes [75–81]. For the present complexes, we considered only bond critical points (BCP) using (3, -1) especially for the interactions zone to elucidate the nature and strength of attractive interactions. In Li@crypt, the Li63 atom shows interaction with O6/O4 and N8 atoms, while Na1 and K63 interact with oxygen (O1, O2, O3, O4, O5, and O6) atoms of cryptand only. Labeled structures with critical points and atoms are given in Figure 3a, while their corresponding parameters from CP are given in supplementary information (Table S6). Generally, greater values of electronic density (ρ_r_) > 0.1 and negative Laplacian of electronic density (∇^2^_ρ_) indicate strong interactions like hydrogen bonding and electrostatic interactions. In contrast, the smaller value of ρ_r_ and positive ∇^2^_ρ_ represents the presence of vdW interactions. The ∇^2^_ρ_ has a positive value for entire interactions involving Li63 with nitrogen and oxygen atoms. Also, ρ_r_ is reduced and becomes less than 0.1, rationalizing the non-covalent nature of the interaction of Li63 inside the cavity. BCP 146 and 107 depict the N7—C54 and O4—C21 interactions having negative values for ∇^2^_ρ_, which disclose the presence of strong interaction (covalent bond). The ∇^2^_ρ_ and energy density (H_r_) values at BCP (3, -1) for Li63 interaction with N8, O1, O4, O6, and O4 are positive, indicating the non-covalent nature of bonding between atoms. Similarly, ∇^2^_ρ_ and H_r_ at BCP (3, -1) are positive for Na1 interactions with O2, O4, O5, O6, and O7 pointing to its weak bonding. Na@crypt where Na1—O6/O7/04/O2/O5 has weak noncovalent interactions with complexant (crypt). For K63—O1, a small value of total electronic density (0.0127 au) is observed. Likewise, total electronic density values are also small for K63-O2, K63-O3, K63-O4, and K63-O5 interactions, justifying the presence of weak interactions or vdW forces. The values of ρ_r_ are less than 0.1 au, and positive values of ∇^2^_ρ_ rationalize the presence of non-covalent interaction. The BCP for N7—K63 and N8—K63 also unfold the weak interaction (noncovalent) from the value of their electronic density and ∇^2^_ρ_. On the other hand, the BCP (98) shows the O2—C36 bond, which has negative ∇^2^_ρ_, while ρ_r_ is higher than 0.2, indicating strong interactions (covalent bond). Figure 3b displays the reduced density gradient (RDG) scatter graph, where the λ_2_ sign is exploited to differentiate between the bonded (λ_2_ <0) and non-bonded (λ_2_ >0) interactions. RDG scatter plots with colour-graded depict the type of interactions between alkali metals and cryptand, where the strong attraction (blue), the weak interaction (green), and the strong repulsion (red) are shown in spikes ranging from -0.035 to + 0.020 au. The blue color side of Figure 3b indicates strong inductive or attractive interactions. One can observe an increase in vdW forces due to interactions between metal (M=Li, Na, and K) and cryptand. The vdW interactions are more prominent in Li@crypt and Na@crypt, which agrees with the previous analysis. Therefore, the existence of noncovalent interaction between M – O/M – N may have a significant impact on triggering the complexes’ optical and NLO response.

**Figure 4.**
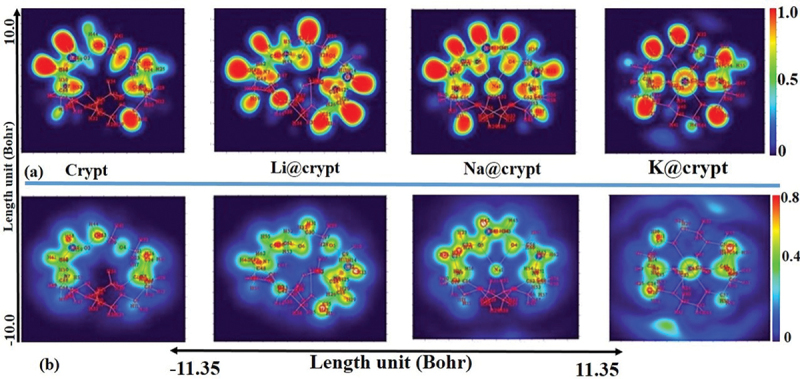
Topological spectra (a) electron localizing function, (b) localized orbital locator (LOL) of crypt and M@crypt electrides using the ωB97×d/def2-tzvp method.

Electron localized function (ELF) and localized orbital locator (LOL) are shown in Figure 4. ELF map is designed in the range of 0.0 to 1.0; however, the delocalized electronic region falls below 0.5. The covalent region (electron-rich) shows high electron density in space where electrons are localized. From the results of color filled ELF map, the red-colored regions of the diaza cryptand [2.2.2] are observed for the hydrogen of the group. Generally, the higher ELF and LOL values, significant localization of electrons, which may be responsible for the existence of covalent bonds, inner shells, or a lone pair of electrons. The synaptic nature of the non-bonding lone pair of electrons on oxygen atoms with alkali metals in M@crypt is displayed in blue color regions in the ELF and LOL map [82]. In the Li@crypt, Li63 has a high ELF value (red region) which indicates that ns^1^ is not completely ionized and still bound with its valence. However, due to the strong interaction of Li63 to the lower side of the crypt, a significant increase in ELF value (red color) can be observed for N8, C12, C18, C15, and hydrogen atoms (H28, 25, 13, and 16). For Na@crypt, ELF value is decreased for Na1, which reveals the ns^1^ valence electrons are more delocalized to ring in composing a diffuse excess electrons model. Na1 is separated by a blue color region, displaying its non-covalent interaction with O2, O4, O5, O6, and O7. In the K@crypt complex, K63 shows further reduced ELF value, while the CH_2_-group hold strong localization (red region). The valence electrons of K are diffused to the entire ring composing an anionic interstitial in cryptand. The Blue color shade can be seen in the LOL spectra of complexes, indicating the delocalized nature of electrons in complexes. The existence of blue circles around the alkali metals unveils the electron deficiency, transferring to complexant (crypt). Mathematically, ELF and LOL exhibit similar chemical mapping because they depend on the kinetic energy density. The ELF function can nicely unveil the location of atomic shells, core binding electrons, and lone pairs electrons in atomic and molecular orbitals. It is a dimensionless quantity, in the range of 0–1, to provide a visual description of the chemical bond for present complexes. Furthermore, ELF includes the Pauli kinetic energy density, while the LOL analysis does not include Pauli repulsion. Mathematically, the ELF can be expressed based on kinetic energy density. In contrast, the increased delocalization of electrons can be observed from Li@crypt to K@crypt. Moreover, the neutral region on LOL (light green) can be seen on bridgehead nitrogen atoms of aza-cryptand, while methylene (CH_2_) moieties of cryptand indicate higher delocalization of electrons.

### UV-visible study

**Figure 5.**
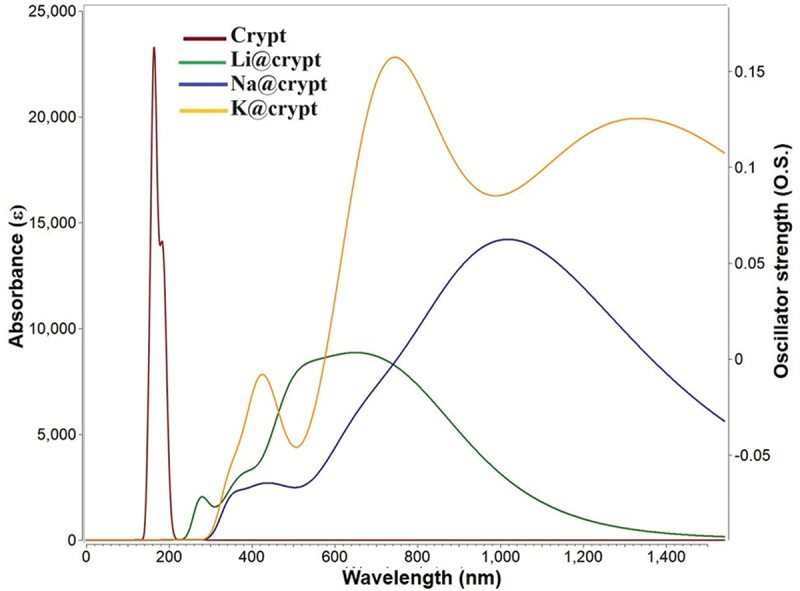
Absorbance spectra of present complexes using the TD- ωB97×d/def2-tzvp method.

For the designed M@crypt complexes, UV-Vis spectral study is exploited using the TD-DFT/ωB97×d/def2tzvp method. In this analysis, the absorbance wavelength (λ), excitation energy (ΔE), and oscillator strength (O.S.) are determined. The contribution of major orbitals during the excitation from HOMO-LUMO is very crucial in the NLO study. Out of 40 excited states, we considered only the crucial state (with significant oscillator strength) for obtaining the excited state parameters (see Table 5). The pure cryptand shows absorbance in the deep ultraviolet region while after complexation with alkali metals, a dramatic increase in absorbance wavelength (bathochromic shift) is seen (see Figure 5). The highest absorbance maxima (λ_max_) is accounted for Na@crypt complex at 1016 nm. Due to an appreciable reduction in excitation energy (ΔE) of Na@crypt, a vital excitation is observed from HOMO→LUMO with a contribution of 43.8%. The increased absorbance maxima can be strongly correlated to the increased atomic number of metals (M). Also, the smaller the excitation energy higher the hyperpolarizability response (vide supra). The oscillator strength (O.S.) of Na@crypt and K@crypt is higher as compared to Li@complex.

**Table 5.** Excitation energy (ΔE in eV), absorbance wavelength (λ_max_ in nm), oscillator strength (O.S. au), and percentage contribution of orbitals during crucial transitions.

TD-DFT parameters from crucial transitions				
Complexes	ΔE	Wavelength (λ_max_)	O.S.	Percentage contributions of orbitals
Crypt	7.73	160	0.14	
Li@Crypt	1.58	783	0.13	HOMO→LUMO (43.8%)
				HOMO→LUMO +1 (32%)
				HOMO→LUMO +4 (4.8%)
Na@Crypt	1.22	1016	0.24	HOMO→LUMO +3 (86%)
				HOMO→LUMO +5 (10%)
				HOMO→LUMO +2 (1.5%)
K@Crypt	1.66	746	0.24	HOMO→LUMO (79%)
				HOMO→LUMO +1 (18.2%)
				HOMO→LUMO +5 (1.3%)

## Conclusion

In summary, we have presented the optical, and NLO properties of designed complexes M@crypt based upon various quantum chemical calculations as well as the non-covalent interactions between the metal center and the cryptand. The designed complexes are similar to molecular electride and could be grouped in the excess electron family. AIMD simulations further confirm their kinetic and thermal stabilities at room temperature. The electronic structure calculations unveil their nature reminiscent of excess electron compounds, where the HOMO-LUMO gap was significantly reduced to 0.38 eV or lower. The C-N and C-O stretching vibrations become more intense after metal coordinating at the cavity of the cryptand. The remarkable value (1.41 × 10^6^ au) of the first-order hyperpolarizability (β_o_) is recorded for Na@crypt electride using ωB97×d/def2-tzvp. The β_HRS_ is recorded up to 2.5 × 10^7^ au. The highest static second-order hyperpolarizability γ_o_ value of 4.96 × 10^9^ au is calculated for K@crypt. γ_o_ increases gradually with the size of alkali metals. Furthermore, the frequency-dependent NLO properties are more responsive than those of static properties. Calculated NLO responses are almost identical regardless of DFT methods. The obtained results are compared to previously reported molecular electride and excess electron compounds. Increasing hyperpolarizability can be affiliated with strong van der Waals interactions, confirmed by QTAIM and RDG analyses. Electron density difference maps are employed to predict the charge transfer and distribution of electronic density after the de-excitation of electrons. The ELF and LOL analysis further disclose the nature of chemical bonds between alkali and complexant. TD-DFT calculations reveal the excited state parameters and absorbance properties. Hence, the examined electrides (as excess electrons compounds) could be a novel addition to optical materials.

## Supplementary Material

Supplemental Material

## Data Availability

The author confirms that data supporting the findings current study are available within the article and in its supporting information. Raw data that supports the findings of this study are available from the corresponding, upon reasonable request.

## Statement of Novelty/Impact statement

Organic electrides M@cryptand [2.2.2] (where M=Li, Na, and K) were examined. Excess electrons and vdW forces play critical roles in promoting the NLO responses.

## References

[1] Anis M, Muley GG, Hakeem A, et al. Exploring the influence of carboxylic acids on nonlinear optical (NLO) and dielectric properties of KDP crystal for applications of NLO facilitated photonic devices. Opt Mater. 2015;46:517–15. doi: 10.1016/j.optmat.2015.04.064

[2] Wu J, Luo AKY, Jen J. High-performance organic second- and third-order nonlinear optical materials for ultrafast information processing. J Mater Chem C. 2020;8(43):15009–15026. doi: 10.1039/D0TC03224G

[3] Luo X, Li Z, Guo Y, et al. Recent progress on new infrared nonlinear optical materials with application prospect. J Solid State Chem. 2019;270:674–685. doi: 10.1016/j.jssc.2018.12.036

[4] Maiman TH. Stimulated optical radiation in ruby. Nature. 1960;187(4736):493–494. doi: 10.1038/187493a0

[5] Gauvin S, Zyss J. Growth of organic crystalline thin films, their optical characterization and application to non-linear optics. J Cryst Growth. 1996;166(1–4):507–527. doi: 10.1016/0022-0248(96)00078-4

[6] E Kalsoom U, Yi R, Qu J, et al. Nonlinear optical properties of CdSe and CdTe core-shell quantum dots and their applications. Front Phys. 2021;9:612070. doi: 10.3389/fphy.2021.612070

[7] Wang L-Y, Shi B-Y, Yao C-B, et al. Size and morphology modulation in ZnO nanostructures for nonlinear optical applications: a review. ACS Appl Nano Mater. 2023;6(12):9975–10014. doi: 10.1021/acsanm.3c01509

[8] Shen HY, He L, Shi PP, et al. Lead-free organic–inorganic hybrid semiconductors and NLO switches tuned by dimensional design. J Mater Chem C. 2021;9(12):4338–4343. doi: 10.1039/D1TC00278C

[9] Notake T, Takeda M, Okada S, et al. Characterization of all second-order nonlinear-optical coefficients of organic N-benzyl-2-methyl-4-nitroaniline crystal. Sci Rep. 2019;9(1):14853. doi: 10.1038/s41598-019-50951-1 31619687 PMC6795865

[10] Zheng Y, Cheng P, Qian X, et al. Self-assembled organic nonlinear optical crystals based on pyridine derived fluorenone. Mater Chem Front. 2023;7(4):698–704. doi: 10.1039/D2QM01173E

[11] Ivanova BB, Spiteller M. Noncentrosymmetric crystals with marked nonlinear optical properties. J Phys Chem A. 2010;114(15):5099–5103. doi: 10.1021/jp1002758 20334443

[12] Jiang X, Zhao S, Lin Z, et al. The role of dipole moment in determining the nonlinear optical behavior of materials: ab initio studies on quaternary molybdenum tellurite crystals. J Mater Chem C. 2014;2(3):530–537. doi: 10.1039/C3TC31872A

[13] Geskin VM, Lambert C, Bredas JL. Origin of high second- and third-order nonlinear optical response in ammonio/borato diphenylpolyene zwitterions: the remarkable role of polarized aromatic groups. J Am Chem Soc. 2003;125(50):15651. doi: 10.1021/ja035862p 14664614

[14] Jeewandara AK, de Silva KMN. Are donor–acceptor self organised aromatic systems NLO (non-linear optical) active? J Mol Struct Theochem. 2004;686(1–3):131–136. doi: 10.1016/j.theochem.2004.08.023

[15] Liu ZB, Zhou ZJ, Li Y, et al. Push–pull electron effects of the complexant in a Li atom doped molecule with electride character: a new strategy to enhance the first hyperpolarizability. Phys Chem Chem Phys. 2010;12(35):10562. doi: 10.1039/c004262e 20614052

[16] Wang S, Dong Y, He C, et al. The role of sp2/sp3 hybrid carbon regulation in the nonlinear optical properties of graphene oxide materials. RSC Adv. 2017;7(84):53643–53652. doi: 10.1039/C7RA10505C

[17] Wang SJ, Wang YF, Cai C. Multidecker sandwich complexes V n Ben n +1 (n = 1, 2, 3) as stronger electron donor relative to ferrocene for designing high-performance organometallic second-order NLO chromophores: Evident layer effect on the first hyperpolarizability and two-dimensional NLO character. J Phys Chem C. 2015;119(10):5589–5595. doi: 10.1021/jp5123272

[18] Maury O, Le Bozec H. Molecular engineering of octupolar NLO molecules and materials based on bipyridyl metal complexes. Acc Chem Res. 2005;38(9):691–704. doi: 10.1021/ar020264l 16171312

[19] Murugan NA, Kongsted J, Rinkevicius Z, et al. Breakdown of the first hyperpolarizability/bond-length alternation parameter relationship. Proc Natl Acad Sci USA. 2010;107(38):16453–16458. doi: 10.1073/pnas.1006572107 20823263 PMC2944713

[20] Shakerzadeh E, Tahmasebi E, Solimannejad M, et al. Tuning the electronic-optical properties of porphyrin-like porous C 24 N 24 fullerene with (Li 3 O) n = (1–5) decoration, a computational study. Appl Organomet Chem. 2019;33(1):1–9. doi: 10.1002/aoc.4654

[21] Ahsin A, Ayub K. Oxacarbon superalkali C_3_X_3_Y_3_ (X = O, S andY = Li, Na,K) clusters as excess electron compounds for remarkable static and dynamic NLO response. J Mol Graph Model. 2021;106:107922. doi: 10.1016/j.jmgm.2021.107922 33984815

[22] Ahsin A, Ali A, Ayub K. Alkaline earth metals serving as source of excess electron for alkaline earth metals to impart large second and third order nonlinear optical response; a DFT study. J Mol Graph Model. 2020;101:107759. doi: 10.1016/j.jmgm.2020.107759 33011558

[23] He HM, Li Y, Sun WM, et al. All-metal electride molecules CuAg@Ca 7 M (M = Be, Mg, and Ca) with multi-excess electrons and all-metal polyanions: molecular structures and bonding modes as well as large infrared nonlinear optical responses. Dalton Trans. 2016;45(6):2656–2665. doi: 10.1039/C5DT04530D 26740006

[24] Zhong R-L, Xu H-L, Li Z-R, et al. Role of excess electrons in nonlinear optical response. J Phys Chem Lett. 2015;6(4):612–619. doi: 10.1021/jz502588x 26262475

[25] Maqbool H, Rafique A, Bhatti IA, et al. Novel endohedrally and exohedrally metals (Li, Na, and K, Ag) doped (15-crown-5) with remarkable electronic, static and dynamic NLO response. Optik. 2022;271:170169. doi: 10.1016/j.ijleo.2022.170169

[26] Ahsin A, Ali A, Ayub K. Transition metals based metalides TM-Janus-TM (where TM=Sc–Zn and Janus=F_6_C_6_H_6_); a theoretical study of nonconventional metalides with excellent static and dynamic nonlinear optical properties. Mater Sci Semicond Process. 2023;162:107506. doi: 10.1016/j.mssp.2023.107506

[27] Sun WM, Ni BL, Wu D, et al. Designing Alkalides with considerable nonlinear optical responses and high stability based on the facially polarized Janus all-cis-1,2,3,4,5,6-Hexafluorocyclohexane. Organometal. 2017;36:3352–3359. doi: 10.1021/acs.organomet.7b00491

[28] Ahsan A, Ayub K. Extremely large nonlinear optical response and excellent electronic stability of true alkaline earthides based on hexaammine complexant. J Mol Liq. 2020;297:111899. doi: 10.1016/j.molliq.2019.111899

[29] Ahsin A, Ayub K. Superalkali-based alkalides Li_3_O@[12-crown-4]M (where M= Li, Na, and K) with remarkable static and dynamic NLO properties; a DFT study. Mater Sci Semicond Process. 2022;138:106254. doi: 10.1016/j.mssp.2021.106254

[30] Ahsan A, Ayub K. Adamanzane based alkaline earthides with excellent nonlinear optical response and ultraviolet transparency. Opt Laser Technol. 2020;129:106298. doi: 10.1016/j.optlastec.2020.106298

[31] Zhu L, Xue K, Hou J. A theoretical study of alkaline-earthides Li(NH_3_)_4_M(M= Be, Mg, Ca) with large first hyperpolarizability. J Mol Model. 2019;25(6):150. doi: 10.1007/s00894-019-4042-3 31065798

[32] Li Z, Yang J, Hou JG, et al. Inorganic electride: theoretical study on structural and electronic properties. J Am Chem Soc. 2003;125(20):6050–6051. doi: 10.1021/ja034020n 12785823

[33] Khaliq F, Mahmood T, Ayub K, et al. Exploring Li4N and Li4O superalkalis as efficient dopants for the Al12N12 nanocage to design high performance nonlinear optical materials with high thermodynamic stability. Polyhedron. 2021;200:115145. doi: 10.1016/j.poly.2021.115145

[34] Hu Q, Tan R, Li J, et al. Highly conductive C_12_A_7_e− electride nanoparticles as an electron donor type promoter to P25 for enhancing photocatalytic hydrogen evolution. J Phys Chem Solids. 2021;149:109810. doi: 10.1016/j.jpcs.2020.109810

[35] Dale SG, Otero-de-la-Roza A, Johnson ER. Density-functional description of electrides. Phys Chem Chem Phys. 2014;16(28):14584. doi: 10.1039/C3CP55533J 24724157

[36] Redko MY, Jackson JE, Huang RH, et al. Design and synthesis of a thermally stable organic electride. J Am Chem Soc. 2005;127(35):12416–12422. doi: 10.1021/ja053216f 16131224

[37] Wajid S, Kosar N, Ullah F, et al. Demonstrating the potential of alkali metal-doped cyclic C_6_O_6_Li_6_ organometallics as electrides and high-performance NLO materials. ACS Omega. 2021;6(44):29852–29861. doi: 10.1021/acsomega.1c04349 34778658 PMC8582031

[38] Kosar N, Zari L, Ayub K, et al. Static, dynamic nonlinear optical (NLO) response and electride characteristics of superalkalis doped star like C_6_S_6_Li_6_. Surf Interfaces. 2002;31:102044. doi: 10.1016/j.surfin.2022.102044

[39] Taschner IS, Walker TL, Schrage BR, et al. Topomeric aza/thia cryptands: synthesis and theoretical aspects of in/out isomerism using n-alkyl bridging. Org Chem Front. 2020;7(9):1164–1176. doi: 10.1039/D0QO00123F

[40] Han Y, Jiang Y, Chen C-F. Cryptand-based hosts for organic guests. Tetrahedron. 2015;71(4):503–522. doi: 10.1016/j.tet.2014.11.006

[41] Dietrich B, Lehn JM, Sauvage JP. Diaza-polyoxa-macrocycles et macrobicycles. Tetrahedron Lett. 1969;10(34):2885–2888. doi: 10.1016/S0040-4039(01)88299-X

[42] Maqsood N, Asif A, Ayub K, et al. DFT study of alkali and alkaline earth metal-doped benzocryptand with remarkable NLO properties. RSC Adv. 2022;12(25):16029–16045. doi: 10.1039/D2RA02209E 35733683 PMC9136961

[43] Huang RH, Faber MK, Moeggenborg KJ, et al. Structure of K^+^(cryptand[2.2.2J) electride and evidence for trapped electron pairs. Nature. 1988;331(6157):599–601. doi: 10.1038/331599a0

[44] Tehan FJ, Barnett BL, Dye JL, et al. Alkali anions. Preparation and crystal structure of a compound which contains the cryptated sodium cation and the sodium anion. J Am Chem Soc. 1974;96(23):7203–7208. doi: 10.1021/ja00830a005

[45] Dye JL. Electrides: early examples of quantum confinement. Acc Chem Res. 2009;42(10):1564–1572. doi: 10.1021/ar9000857 19645438

[46] Cao J, Li F, Xia W, et al. Interactions in bimolecular reactions. Chin J Chem Phys. 2019;32(2):157–166. doi: 10.1063/1674-0068/cjcp1901007

[47] Shen Z, Ma H, Zhang C, et al. Dynamical importance of van der Waals saddle and excited potential surface in C(^1^D)+D2 complex-forming reaction. Nat Commun. 2017;8(1):14094. doi: 10.1038/ncomms14094 28094253 PMC5247604

[48] Frisch MJ, Trucks GW, Schlegel HB, et al. Gaussian 16, Revision A.03. Wallingford CT: Gaussian, Inc; 2016.

[49] Neese F, Wennmohs F, Becker U, et al. The ORCA quantum chemistry program package. J Chem Phys. 2020;152(22):224108. doi: 10.1063/5.0004608 32534543

[50] Chai J-D, Head-Gordon M. Long-range corrected hybrid density functionals with damped atom–atom dispersion corrections. Phys Chem Chem Phys. 2008;10(44):6615–6620. doi: 10.1039/b810189b 18989472

[51] Pérez P, Toro-Labbé A, Contreras R. Solvent effects on electrophilicity. J Am Chem Soc. 2001;123(23):5527–5531. doi: 10.1021/ja004105d 11389635

[52] Chermette H. Chemical reactivity indexes in density functional theory. J Comput Chem. 1999;20(1):129–154. doi: 10.1002/(SICI)1096-987X(19990115)20:1<129:AID-JCC13>3.0.CO;2-A

[53] Castet F, Rodriguez V, Pozzo JL, et al. Design and characterization of molecular nonlinear optical switches. Acc Chem Res. 2013;46(11):2656. doi: 10.1021/ar4000955 23865890

[54] Asselberghs I, Flors C, Ferrighi L, et al. Second-harmonic generation in GFP-like proteins. J Am Chem Soc. 2008;130(46):15713–15719. doi: 10.1021/ja805171q 18950177

[55] Cukrowski I, de Lange JH, Adeyinka AS, et al. Evaluating common QTAIM and NCI interpretations of the electron density concentration through IQA interaction energies and 1D cross-sections of the electron and deformation density distributions. Comput Theory Chem. 2015;1053:60–76. doi: 10.1016/j.comptc.2014.10.005

[56] Lu T, Chen, FW. Multiwfn: A multifunctional wavefunction analyzer. J Comput Chem. 2012;33(5):580–592. doi: 10.1002/jcc.22885 22162017

[57] Fuster F, Sevin A, Silvi B. Topological analysis of the electron localization function (ELF) applied to the electrophilic aromatic substitution. J Phys Chem A. 2000;104(4):852–858. doi: 10.1021/jp992783k

[58] Clements RJ, Womack JC, Skylaris CK. Electron localisation descriptors in ONETEP: A tool for interpreting localisation and bonding in large-scale DFT calculations. Electron Struct. 2020;2(2):027001. doi: 10.1088/2516-1075/ab8d19

[59] Tkachenko NV, Sun Z-M, Boldyrev AI. Record low ionization potentials of Alkali metal complexes with crown ethers and cryptands. Chemphyschem. 2019;20(16):2060–2062. doi: 10.1002/cphc.201900422 31184431

[60] Chekhlov AN. Synthesis and crystal structure of (2.2.2-cryptand)potassium nitrate hydrate. Russ J Coord Chem. 2006;32(1):5–9. doi: 10.1134/S1070328406010027

[61] Kosar N, Zari L, Ayub K, et al. NLO properties and electride characteristics of superalkalis doped all-cis-1. Optik. 2022;271(271):170139. doi: 10.1016/j.ijleo.2022.170139

[62] Konarev DV, Khasanov SS, Ishikawa M, et al. Metallic conductivity versus charge disproportionation in C_60_ complexes with noninteger average charges on fullerene. Chem Select. 2016;1(2):323–330. doi: 10.1002/slct.201500021

[63] Rehm E, Boldyrev AI, Schleyer PVR. Ab initio study of superalkalis. First ionization potentials and thermodynamic stability. Inorg Chem. 1992;31(23):4834–4842. doi: 10.1021/ic00049a022

[64] Ahsin A, Jadoon T, Ayub K. M@[12-crown-4] and M@15-crown-5] where (M=li, Na, and K); the very first examples of non-conventional one alkali metal-containing alkalides with remarkable static and dynamic NLO response. Phys E Low-Dimens Syst Nanostruct. 2022;140:115170. doi: 10.1016/j.physe.2022.115170

[65] Ahsin A, Shah AB, Ayub K. Germanium-based superatom clusters as excess electron compounds with significant static and dynamic NLO response; a DFT study. RSC Adv. 2022;12(1):365–377. doi: 10.1039/D1RA08192F PMC8978613 35424493

[66] Wang J-J, Zhou Z-J, Bai Y, et al. A new strategy for simultaneously enhancing nonlinear optical response and electron stability in novel cup–saucer + –cage − -shaped sandwich electride molecules with an excess electron protected inside the cage. Dalton Trans. 2015;44(9):4207–4214. doi: 10.1039/C4DT03282A 25627170

[67] Ahsin A, Ejaz I, Sarfaraz S, et al. Polaron formation in conducting polymers: a novel approach to designing materials with a larger NLO response. ACS Omega. 2024;9(12):14043–14053. doi: 10.1021/acsomega.3c09468 38559943 PMC10976349

[68] Ullah F, Kosar N, Ayub K, et al. Superalkalis as a source of diffuse excess electrons in newly designed inorganic electrides with remarkable nonlinear response and deep ultraviolet transparency: A DFT study. Appl Surf Sci. 2019;483:1118–1128. doi: 10.1016/j.apsusc.2019.04.042

[69] Kosar N, Zari L, Ayub K, et al. Giant NLO response and ultraviolet transparency of superalakalis decorated C_6_O_6_Li_6_ complexes; a DFT perspective. Phys Scr. 2023;98(6):98 65909. doi: 10.1088/1402-4896/accf4b

[70] Wang Y-F, Huang J, Jia L, et al. Theoretical investigation of the structures, stabilities, and NLO responses of calcium-doped pyridazine: alkaline-earth-based alkaline salt electrides. J Mol Graph Model. 2014;47:77–82. doi: 10.1016/j.jmgm.2013.11.003 24361791

[71] Williams MD, Ford JS, Andrews DL. Hyper-Rayleigh scattering in centrosymmetric systems. J Chem Phys. 2015;143(12):124301. doi: 10.1063/1.4931584 26429005

[72] Maina MR, Okamoto Y, Hamada K, et al. Effects of superposition of 532 nm and 1064 nm wavelengths in copper micro-welding by pulsed Nd: YAG laser. J Mater Process Technol. 2022;299:117388. doi: 10.1016/j.jmatprotec.2021.117388

[73] Abel S, Eltes F, Ortmann JE, et al. Large pockels effect in micro- and nanostructured barium titanate integrated on silicon. Nat Mater. 2019;18(1):42–47. doi: 10.1038/s41563-018-0208-0 30420671

[74] Shi Q, Dong L, Wang Y. Evaluating refractive index and birefringence of nonlinear optical crystals: classical methods and new developments. Chines J Struct Chem. 2023;42(1):100017. doi: 10.1016/j.cjsc.2023.100017

[75] Lu Q, Bian W. The decay of dispersion interaction and its remarkable effects on the kinetics of activation reactions involving alkyl chains. J Phys Chem Lett. 2023;14(47):10642–10647. doi: 10.1021/acs.jpclett.3c02925 38031665

[76] Yang X, Ma H, Lu Q, et al. Efficient method for numerical calculations of molecular vibrational frequencies by exploiting sparseness of Hessian matrix. J Phys Chem A. 2023;128(15):3024–3032. doi: 10.1021/acs.jpca.3c07645 PMC11033861 38484711

[77] Li F, Yang X, Liu X, et al. An Ab initio neural network potential energy surface for the dimer of formic acid and further quantum tunneling dynamics. ACS Omega. 2023;8(19):17296–17303. doi: 10.1021/acsomega.3c02169 37214673 PMC10193396

[78] Li F, Liu X, Yang X, et al. Quantum dynamics calculations on isotope effects of hydrogen transfer isomerization in formic acid dimer. Chin J Chem Phys. 2023;36(5):545–552. doi: 10.1063/1674-0068/cjcp2301009

[79] Wu Y, Cao J, Ma H, et al. Conical intersection–regulated intermediates in bimolecular reactions: Insights from C(^1^D) + HD dynamics. Sci Adv. 2019;5(4):2375–2548. doi: 10.1126/sciadv.aaw0446 PMC6486230 31032418

[80] Cao J, Wu Y, Bian W. Ring polymer molecular dynamics of the C(^1^D)+H_2_ reaction on the most recent potential energy surfaces. Chin J Chem Phys. 2021;34(6):833–842. doi: 10.1063/1674-0068/cjcp2110197

[81] Zhang C, Fu M, Shen Z, et al. Global analytical ab initio ground-state potential energy surface for the C(^1^D)+H_2_ reactive system. J Chem Phys. 2014;140(23):234301–234310. doi: 10.1063/1.4881896 24952535

[82] Michalski M, Gordon AJ, Berski S. Topological analysis of the electron localisation function (ELF) applied to the electronic structure of oxaziridine: the nature of N-O bond. Struct Chem. 2019;30(6):2181–2189. doi: 10.1007/s11224-019-01407-9

