# Supplementary material for: Theoretically designed M@diaza[2.2.2]cryptand complexes: the role of non-covalent interactions in promoting NLO properties of organic electrides
Source: Sci Technol Adv Mater. 2024 May 20;25(1):2357064. doi: 10.1080/14686996.2024.2357064 (PMC11149575; doi:10.1080/14686996.2024.2357064)
Supplement: Supplemental Material [file TSTA_A_2357064_SM9064.docx]

**Supplementary information for:**

Theoretically designed M@diaza[2.2.2]cryptand complexes: the role of non-covalent interactions in promoting NLO properties of organic electrides

Atazaz Ahsin^1,3^ Aamna Qamar^2,3^ Qing Lu^1*^ Wensheng Bian^1,3*^

^1^Beijing National Laboratory for Molecular Sciences, Institute of Chemistry, Chinese Academy of Sciences, Beijing 100190, China,

^2^Beijing National Laboratory for Molecular Sciences, State Key Laboratory of Polymer Physics and Chemistry, Chinese Academy of Sciences, Beijing 100190, China

^3^School of Chemical Sciences, University of Chinese Academy of Sciences, Beijing 100049, China

Correspondence^*^:)，qinglu@iccas.ac.cn (Q. L), bian@iccas.ac.cn (W. B)

**Table of Contents**

[Table S1: Optimized geometrical parameters S3](#_Toc165758721)

[Table S2: The calculated and experimentally reported vibrational frequencies of pure (cryptand) and designed complexes S4](#_Toc165758722)

[Discussion on electride nature and comparison with reported similar system S5](#_Toc165758723)

[Table S3: The comparison of electronic properties with reported similar complexes having electrides nature and simply excess electrons compounds. S5](#_Toc165758724)

[Table S4: Polarizability (α](#_Toc165758725)_[o](#_Toc165758725)_ [au), hyperpolarizability (β](#_Toc165758725)_[o](#_Toc165758725)_ [au), and projection of hyperpolarizability on dipole moment vector (β](#_Toc165758725)_[vec](#_Toc165758725)_ [au). S7](#_Toc165758725)

[Table S5: Comparison of static first and second hyperpolarizability (in au) of designed complexes with reported electrides and excess electrons compounds. S7](#_Toc165758726)

[Table S6: QTAIM and its parameters at BCP (3,-1) of M@crypt complexes S8](#_Toc165758727)

[Discussion of EDDM and DOS study of complexes S9](#_Toc165758728)

[Figure S1: AIMD analysis of complexes S10](#_Toc165758729)

[Figure S2: Drift energy (K) versus time of pure and M@crypt complexes. S10](#_Toc165758730)

[Figure S3: Snapshots of pure and designed complexes. S11](#_Toc165758731)

[Figure S4: Molecular orbitals from EDDM analysis of designed electrides complexes S11](#_Toc165758732)

[Figure S5: projected density of states (PDOS) spectra of electrides S12](#_Toc165758733)

Table S1: Optimized geometrical parameters, bond distances are (in Å)

| Calculated at the ωB97xd/def2-tzvp | | Experimental | |
| --- | --- | --- | --- |
| Diaza(2.2.2)cryptand (crypt) | |  |  |
| Bond type | magnitude |  |  |
| C-C in CH_2_-CH_2_ | 1.50 | C-C | 1.48 |
| C-H | 1.10 | C-H | 1.09 |
| C-O1/O2/O3/O4/O5/O6 | 1.40 | C-O | 1.41 |
| N8-C12/18/24  N7-C54/C48/C60 | 1.44 | N-C | 1.46 |
| Li@crypt | |  |  |
| C-C | 1.51 | C-C |  |
| C-H | 1.09 | C-H |  |
| C-O | 1.40 | C-O |  |
| N-C | 1.45 | N-C |  |
| Li63-N7 | 3.95 |  |  |
| Li63-N8 | 2.46 |  |  |
| Li63-O3/O4/O3/O6 | 4.5/2.34/2.70/2.70 |  |  |
| Na@crypt | | | |
| Na1-N8/N9 | 3.19 |  |  |
| Na1-O4/O5 | 2.488 |  |  |
| Na1-O2/O3 | 2.89/2.82 |  |  |
| Na1-O7/O6 | 2.89/2.82 |  |  |
| K@crypt | | [K(Crypt-222)](NO3) 1.5H2O [1] | |
| C-C | 1.51 | C-C | 1.49 |
| C-H | 1.09 | C-H | 1.08 |
| C-O | 1.40 | C-O | 1.41 |
| N-C | 1.45 | N-C | 1.46 |
| K63-N7/N8 | 3.07 | K63-N7/N8 | 2.94/2.97 |
| K63-O1/O2 | 2.83 | K63-O3/O4 | 2.83/2.78 |
| K63-O3/O4  K-O | 2.82/2.83  2.83 [50]^b^ | K63-O3/O4 | 2.77/2.87 |

[b] Average reported distance between oxygen atoms and K-metal in K[2.2.2]

Table S2: The calculated and experimentally reported vibrational frequencies of pure (cryptand) and designed complexes using the ωB97xd/def2-tzvp**.** The calculated stretching vibration are corrected by multiplying 0.96 in order to get comparison with experimental values

|  | **Frequency (cm^-1^)** | |  |  |
| --- | --- | --- | --- | --- |
|  | Calculated at the ωB7XD/def2-tzvp | Experimental [3] | Group | Vibration |
| **Cryptand** | 2812 | 2790-2877 | C-H | Stretching |
|  | 2896 |  | C-H | Stretching |
|  | 2981 |  | C-H | Stretching |
|  | 1523 | 1452 | C-H | Bending |
|  | 1173-1181 | 1295 | C-N | Stretching |
|  | 1158.72 | 1111-1127 | C-O | Stretching |
|  | 1047-1063 | 1038-1078 | C-C | Stretching |
| **Li@crypt** |  |  |  |  |
|  | 2782-3004 |  | C-H | Stretching |
|  | 1274- 1533 |  | C-H | Bending |
|  | 1168-1181 |  | C-N | Stretching |
|  | 1109-1156 |  | C-O | Stretching |
|  | 1067 |  | C-C | Stretching |
| **Na@crypt** |  |  |  |  |
|  | 2826-2990 |  | C-H | Stretching |
|  | 1224-1495 |  | C-H | Bending |
|  | 1157-1176 |  | C-N | Stretching |
|  | 1090-1157 |  | C-O | stretching |
|  | 1059-1075 |  | C-C | Stretching |
| **K@crypt** | 2833- 2998 |  | C-H | Stretching |
|  | 1275-1526 |  | C-H | Bending |
|  | 1168-1172 |  | N-C | Stretching |
|  | 1173-1157 |  | C-O | Stretching |
|  | 1059-1066 |  | C-C | Stretching |

# **Discussion on electride nature and comparison with reported similar system**

The energy of the highest occupied molecular orbitals (E_HOMO_), lowest unoccupied molecular orbitals (E_LUMO_), and HOMO-LUMO gaps (E_H-L_) have significant roles in determining the reactivity of molecules. The HOMO energies of designed complexes are -2.12, -1.17, and -1.46 eV, while LUMO energies lie in the range of -2.59, 1.55, and 2.21 eV for **Li@crypt**, **Na@crypt**, and **K@crypt** (Table 1). The HOMO energies are increasing from Li to K with increased atomic number, while a significant reduction in HOMO-LUMO gaps is observed for complexes as compared to pure complexant. The E_H-L_ gaps are from 0.47, 0.38, and 1.06 eV, respectively, for **Li@crypt**, **Na@crypt**, and **K@crypt,** where the lowest value is obtained for **Na@crypt** complex. Overall, the values are significantly reduced as compared to E_H_-_L_ of the pristine crypt (4.46 eV). The contribution of HOMO-1 and the energy difference HOMO-1 to LUMO are also given in Figure 2. The obtained HOMO-LUMO gaps of present complexes are significantly lower than those of superalkali@F_6_C_6_H_6_ and M(BCM) complexes at the ωB97xd/def2tzvp method [4][5].

Table S3: The comparison of electronic properties with reported similar complexes having electrides nature and simply excess electrons compounds.

| **Alkali and alkaline doped benzocryptand M(BC)M at B3LYP/6-31(G)** [4] | | | | |
| --- | --- | --- | --- | --- |
|  | **E_int_ (kcal/mol)** | **E_H-L_ (eV)** | **VIP (eV)** | **Ƞ (eV)** |
| **Li(BC)Be** | −9.72 | 1.21 | 1.97 | 0.61 |
| **Na(BC)Be** | −9.73 | 1.14 | 1.96 | 0.57 |
| **K(BC)Be** | −0.43 | 2.23 | 2.95 | 1.12 |
| **Superalkali@ C_6_S_6_Li_6_ at the  ωb97xd/6–31+G(d,p)** [6] | | | | |
| **Li_3_O@C_6_S_6_Li_6_** | −45.56 | 1.69 | 2.20 |  |
| **Na_3_O@C_6_S_6_Li_6_** | −42.86 | 1.68 | 2.18 |  |
| **K_3_O@C_6_S_6_Li_6_** | −43.35 | 2.33 | 2.63 |  |
| **Superalkalis doped F_6_C_6_H_6_ at the ωb97xd/6–31+G (d,p)** [5] | | | | |
| **Li_2_F@C_6_F_6_H_6_** | -16.54 | 2.50 | 3.02 |  |
| **Li_2_Cl@C_6_F_6_H_6_** | -14.57 | 3.78 | 3.87 |  |
| **Li_2_Br@C_6_F_6_H_6_** | -16.52 | 3.41 | 3.75 |  |
| **Na_2_F@C_6_F_6_H_6_** | -13.57 | 2.42 | 2.91 |  |
| **Na_2_Br@C_6_F_6_H_6_** | -13.98 | 3.58 | 3.81 |  |
| **K_2_F@C_6_F_6_H_6_** | -14.28 | 1.97 | 2.49 |  |
| **K_2_Cl@C_6_F_6_H_6_** | -14.51 | 3.48 | 3.48 |  |

# Table S4: Polarizability (α_o_ au), hyperpolarizability (β_o_ au), and projection of hyperpolarizability on dipole moment vector (β_vec_ au)**.**

|  | **ωB97xd/def2-tzvp** | | | **CAM-B3LYP/def2-tzvp** | | | **M062X/def2-tzvp** | | |
| --- | --- | --- | --- | --- | --- | --- | --- | --- | --- |
|  | **α_o_** | **β_o_** | **β_vec_** | **α_o_** | **β_o_** | **β_vec_** | **α_o_** | **β_o_** | **β_vec_** |
| **Crypt** | 2.54×10^2^ | 1.02×10^2^ | 6.489 | 2.53×10^2^ | 0.037 | 0.009 | 2.5×10^2^ | 16.729 | 15.287 |
| **Li@crypt** | 3.47×10^2^ | 5.32×10^3^ | 3.59×10^3^ | 3.56×10^2^ | 3.96×10^3^ | 9.62×10^2^ | 3.5×10^2^ | 3.9×10^3^ | 9.6×10^2^ |
| **Na@crypt** | 8.66×10^2^ | 1.41×10^6^ | 1.19×10^6^ | 1.41×10^3^ | 4.18×10^5^ | 4.18×10^5^ | 5.3×10^2^ | 3.2×10^4^ | 3.2×10^4^ |
| **K@crypt** | 9.30×10^2^ | 9.83×10^5^ | 2.96×10^5^ | 7.75×10^3^ | 2.93×10^3^ | 2.93×10^2^ | 1.1×10^3^ | 2.5×10^6^ | 3.1×10^5^ |

# Table S5: Comparison of static first and second hyperpolarizability (in au) of designed complexes with reported electrides and excess electrons compounds.

| **Our studied Complexes M@Crypt (where M=Li, Na, and K)** | | |
| --- | --- | --- |
| **Complexes** | **β_o_** (**ωb7xd/def2-tzvp)** | **γ_o_** |
| **Crypt** | 1.02×10^2^ | **-** |
| **Li@crypt** | 5.32×10^3^ | 1.72×10^10^ |
| **Na@crypt** | 1.41×10^6^ | 2.57×10^11^ |
| **K@crypt** | 9.83×10^5^ | 2.87×10^4^ |
| **C_6_S_6_Li_6_@Superalkali at ωb97xd/6–31+G(d,p) level**[6] | | |
| **C_6_S_6_Li_6_** | 2.65 |  |
| **Li_3_O@C_6_S_6_Li_6_** | 4.54×10^6^ | 2.06×10^10^ |
| **Na_3_O@C_6_S_6_Li_6_** | 2.09×10^6^ | 9.62×10^12^ |
| **K_3_O@C_6_S_6_Li_6_** | 2.98×10^5^ | 2.25×10^8^ |
| **Superalkali@C_6_F_6_H_6_ at ωb97xd/6–31+G (d,p) level of theory [5]** | | |
| **Li_2_F@C_6_F_6_H_6_** | 9.32 × 10^4^ | 3.44 × 10^7^ |
| **Li_2_Cl@C_6_F_6_H_6_** | 3.26 × 10^4^ | 5.69 × 10^6^ |
| **Li_2_Br@C_6_F_6_H_6_** | 8.49 × 10^3^ | 5.07 × 10^8^ |
| **Na_2_F@C_6_F_6_H_6_** | 1.68 × 10^6^ | 3.35 × 10^7^ |
| **Na_2_Br@C_6_F_6_H_6_** | 1.77 × 10^4^ | 3.18 × 10^4^ |
| **K_2_F@C_6_F_6_H_6_** | 4.07 × 10^5^ | 1.52 × 10^8^ |
| **K_2_Cl@C_6_F_6_H_6_** | 3.89 × 10^4^ | 1.39 × 10^7^ |
| **Li_3_@C_60_ and Li_3_@B_40_ electrides** [7] | | |
| **Li_3_@C_60_** | 129.4 | 3.6 × 10^5^ |
| **Li_3_@B_40_** | 79.9 | 2.1 × 10^5^ |
| **Alkali and Alkaline metals doped M(BC)M benzocryptand** [4] | | |
| **Li(BC)Be** | 2.0 89× 10^4^ |  |
| **Na(BC)Be** | 2.35  × 10^4^ |  |
| **K(BC)Be** | 3.39  × 10^3^ |  |

# Table S6: QTAIM and its parameters at BCP (3,-1) of M@crypt complexes: value are in a.u

| **Li@crypt** | | | | | | |
| --- | --- | --- | --- | --- | --- | --- |
| CP | Interactions | ρ_r_ | ∇^2^_ρ_ | G_r_ | V_r_ | H_r_ |
| 120 | N8—Li63 | 0.0098 | 0.0578 | 0.1131 | -0.0081 | 0.0031 |
| 109 | O1-- Li63 | 0.0123 | 0.9642 | 0.0185 | -0.0561 | 0.0067 |
| 116 | 04-- Li63 | 0.9011 | 0.0634 | 0.0119 | -0.0080 | 0.0039 |
| 141 | O6-- Li63 | 0.0117 | 0.0845 | 0.0160 | -0.0116 | 0.0050 |
| 127 | O4—H53 | 0.869 | 0.0337 | 0.0079 | -0.0052 | 0.0015 |
| 93 | O1--04 | 0.0066 | 0.02543874 | 0.0050 | -0.0037 | 0.0013 |
| 131 | O4--06 | 0.0087 | 0.0972156 | 0.0079 | -0.0067 | 0.0019 |
| 146 | N7—C54 | 0.2748 | -0.7203550 | 0.1186 | -0.4174 | -0.2987 |
| 107 | O4—C21 | 0.2620 | -0.5610097 | 0.2283 | -0.5977 | -0.3686 |
| **Na@crypt** | | | | | | |
| 99 | O7-Na1 | 0.4782 | 0.2578 | 0.0048 | -0.0032 | 0.1612 |
| 123 | O4-NA1 | 0.0130 | 0.0048 | 0.0160 | -0.0112 | 0.0048 |
| 93 | O6-Na1 | 0.5327 | 0.0303 | 0.0056 | -0.3702 | 0.0019 |
| 105 | O2-Na1 | 0.4779 | 0.0257 | 0.0048 | -0.0032 | 0.0016 |
| 120 | O5-Na1 | 0.0138 | 0.0834 | 0.0160 | -0.0111 | 0.0048 |
| **K@crypt** | | | | | | |
| 104 | O1--K63 | 0.0127 | 0.0640 | 0.0127 | -0.0094 | 0.3296 |
| 106 | O2--K63 | 0.0127 | 0.0640 | 0.0127 | -0.0094 | 0.0032 |
| 100 | O3--K63 | 0.0128 | 0.0487 | 0.0128 | 0.0095 | 0.0033 |
| 95 | O4--K63 | 0.0128 | 0.0646 | 0.0128 | -0.0095 | 0.0033 |
| 128 | O5--K63 | 0.0128 | 0.0648 | 0.0128 | -0.0095 | 0.0033 |
| 130 | O6--K63 | 0.0128 | 0.0647 | 0.0128 | -0.0095 | 0.3003 |
| 111 | N8-K63 | 0.0100 | 0.0403 | 0.0088 | -0.0061 | 0.0019 |
| 118 | N8-K63 | 0.0100 | 0.0403 | 0.0081 | -0.0061 | 0.0019 |
| 145 | O6-H37 | 0.0047 | -0.0019 | 0.0007 | -0.00003 | -0.0004 |
| 98 | O2—C36 | 0.2650 | -0.5758 | 0.2316 | -0.6073 | -0.3756 |

# **Discussion of EDDM and DOS study of complexes**

An electronic density difference map (EDDM) is used to predict the charge transferability and distribution of orbital after the relaxation of the electron from an excited state to a ground state (ρ_excited_ − ρ_ground_). From EDDM pictures, distinct colors (purple, cyan-blue) depict various orbital densities in different regions of complexant. The 3D-mapped EDDM spectra are given in Figure S4. Purple is used to represent the nucleophilic zone, where electron density drops with charge transfer, and cyan-blue is used to represent the electrophilic region, where electron density increases with charge transfer. For **Li@crypt,** orbital density is higher in the lower region of the complex, which might be due to strong interaction with N7 and significant charge transfer. Likewise, the complex **Na@crypt** shows a distribution of electron density close to Na-metals in the middle of the cavity. In **K@Crypt**, the K-metal adsorbed in the center of the complexant, and the charge transfer effect is uniform throughout the cryptand.

The plotted TDOS of pure (crypt) is shown in Figure S6, while the projected density of state (PDOS) is shown in Figure S5. For the pristine crypt, the TDOS spectra are plotted between an energy range of -20 to 20 eV, and the HOMO line appears near -7 eV. The HOMO-LUMO gap is also wider for pristine crypt. Likewise, in PDOS spectra, the contribution of alkali metals as fragments displayed a crucial role in narrowing the HOMO-LUMO gaps. The red color indicates the contribution from the main complexant, while the blue color line shows the contribution to the density of states from alkali metals.


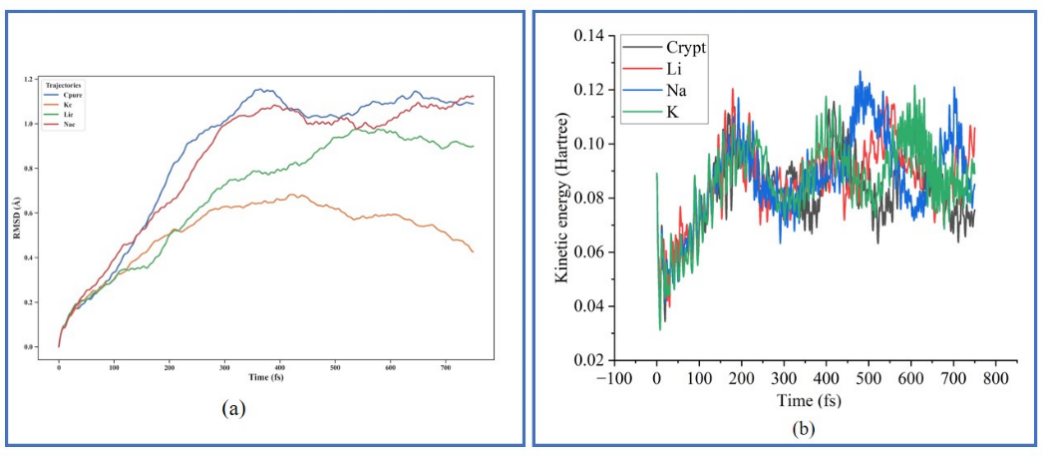


# Figure S1: AIMD analysis of complexes. (a) root-mean-square deviation (RMSD), (b) Kinetic energy of crypt and M@crypt complexes at 300 K and 750 (time fs) using the B3LYP-D3/def2-SVP method.

# Figure S2: Drift energy (K) versus time of pure and M@crypt complexes at 300 K during the ab-initio molecule dynamic (AIMD) simulation using the B3LYP-D3/def2-SVP method.


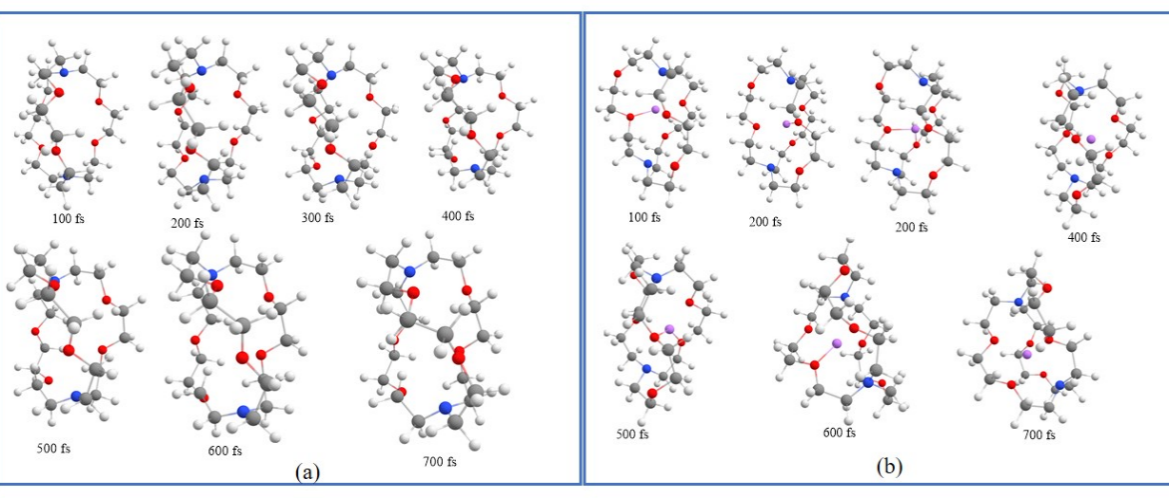


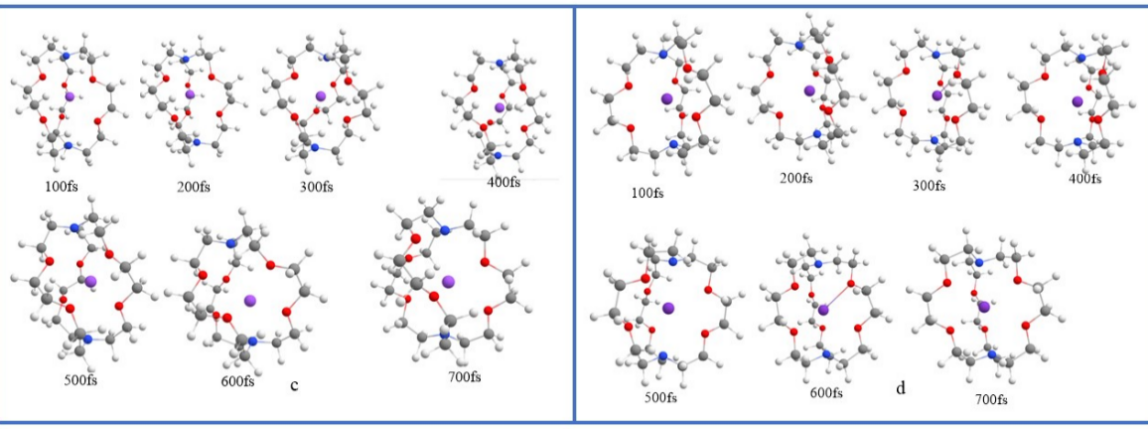


# Figure S3: Snapshots of pure and designed complexes during 750 fs using 1500 steps**.** (a) shows the pure (diaza cryptand[2.2.2]), (b) Li@crypt, (c) Na@crypt, (d) K@crypt during AIMD study.

**
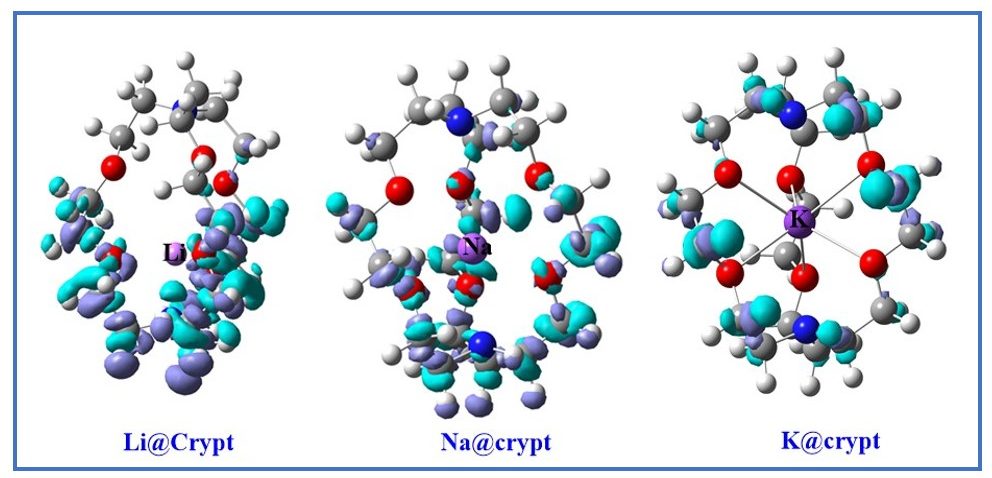
**

# Figure S4: Molecular orbitals from EDDM analysis of designed electrides complexes

**Figure S5: projected density of states (PDOS) spectra of electrides**: blue curve show the contribution of metals to orbitals while red line for complexant. The black curve is representing total density of states using the ωB97xd/def2-tzvp method

**References**

[1] A.N. Chekhlov, Synthesis and crystal structure of (2.2.2-cryptand)potassium nitrate hydrate, Russ. J. Coord. Chem. Khimiya. 2006; 32: 5–9.

[2] N. V Tkachenko, Z.-M. Sun, A.I, et al. Record Low Ionization Potentials of Alkali Metal Complexes with Crown Ethers and Cryptands, ChemPhysChem. 2019; 20: 2060–2062.

[3] D. V Konarev, S.S. Khasanov, M. Ishikawa, et al. Metallic conductivity versus charge disproportionation in C60 complexes with noninteger average charges on fullerene, ChemistrySelect. 2016; 1: 323–330.

[4] N. Maqsood, A. Asif, K. Ayub, et al. DFT study of alkali and alkaline earth metal-doped benzocryptand with remarkable NLO properties, RSC Adv. 2022; 12: 16029–16045.

[5] N. Kosar, L. Zari, K. Ayub, et al. NLO properties and electride characteristics of superalkalis doped all-cis-1,2,3,4,5,6-hexafluorocyclohexane complexes, Optik. 2022; 271: 170139.

[6] N. Kosar, L. Zari, K. Ayub,et al. Static, dynamic nonlinear optical (NLO) response and electride characteristics of superalkalis doped star like C_6_S_6_Li_6_, Surfaces and Interfaces. 2022; 31: 102044.

[7] P. Das, P.K. Chattaraj, Comparison Between Electride Characteristics of Li_3_@B_40_ and Li_3_@C_60_ , Front. Chem. 2021; 9: [638581](https://doi.org/10.3389/fchem.2021.638581)
